# Next-generation ELISA diagnostic assay for Chagas Disease based on the combination of short peptidic epitopes

**DOI:** 10.1371/journal.pntd.0005972

**Published:** 2017-10-09

**Authors:** Juan Mucci, Santiago J. Carmona, Romina Volcovich, Jaime Altcheh, Estefanía Bracamonte, Jorge D. Marco, Morten Nielsen, Carlos A. Buscaglia, Fernán Agüero

**Affiliations:** 1 Instituto de Investigaciones Biotecnológicas (IIB)–Instituto Tecnológico de Chascomús (INTECH), Universidad Nacional de San Martín (UNSAM)–Consejo Nacional de Investigaciones Científicas y Técnicas (CONICET), San Martín, Buenos Aires, Argentina; 2 Servicio de Parasitología y Chagas, Hospital de Niños Ricardo Gutierrez, Ciudad Autónoma de Buenos Aires, Argentina; 3 Instituto de Patología Experimental, Facultad de Ciencias de la Salud, Universidad Nacional de Salta (UNSa)–Consejo Nacional de Investigaciones Científicas y Técnicas (CONICET), Salta, Argentina; 4 Department of Bio and Health Informatics, Technical University of Denmark, DK Lyngby, Denmark; Instituto de Ciências Biológicas, Universidade Federal de Minas Gerais, BRAZIL

## Abstract

Chagas Disease, caused by the protozoan *Trypanosoma cruzi*, is a major health and economic problem in Latin America for which no vaccine or appropriate drugs for large-scale public health interventions are yet available. Accurate diagnosis is essential for the early identification and follow up of vector-borne cases and to prevent transmission of the disease by way of blood transfusions and organ transplantation. Diagnosis is routinely performed using serological methods, some of which require the production of parasite lysates, parasite antigenic fractions or purified recombinant antigens. Although available serological tests give satisfactory results, the production of reliable reagents remains laborious and expensive. Short peptides spanning linear B-cell epitopes have proven ideal serodiagnostic reagents in a wide range of diseases. Recently, we have conducted a large-scale screening of *T*. *cruzi* linear B-cell epitopes using high-density peptide chips, leading to the identification of several hundred novel sequence signatures associated to chronic Chagas Disease. Here, we performed a serological assessment of 27 selected epitopes and of their use in a novel multipeptide-based diagnostic method. A combination of 7 of these peptides were finally evaluated in ELISA format against a panel of 199 sera samples (Chagas-positive and negative, including sera from Leishmaniasis-positive subjects). The multipeptide formulation displayed a high diagnostic performance, with a sensitivity of 96.3% and a specificity of 99.15%. Therefore, the use of synthetic peptides as diagnostic tools are an attractive alternative in Chagas’ disease diagnosis.

## Author summary

Chagas disease, caused by the parasite *Trypanosoma cruzi*, is a life-long and debilitating illness of major significance throughout Latin America, and an emergent threat to global public health. Diagnostic tests are key tools to support disease surveillance, and to ultimately help stop transmission of the parasite. However currently available diagnostic methods have several limitations. Identification of novel biomarkers with improved diagnostic characteristics is a main priority. Recently, we conducted a large-scale screening looking for new *T*. *cruzi* antigens using short peptides displayed on a solid support at high-density. This led to the identification of several hundred novel antigenic epitopes. In this work we validated the serodiagnostic performance of 27 of these against an extended panel of human serum samples. Based on this analysis, we developed a proof-of-principle multiplex diagnostic kit by combining different validated reactive peptides. Overall, our data support the applicability of high-density peptide microarrays for the rapid identification and mapping epitopes that could be readily translated into novel and useful tools for diagnosis of Chagas disease.

## Introduction

Chagas disease is a major health and economic problem in Latin America, for which no vaccine or appropriate drugs for large-scale public health interventions are yet available [1]. It is caused by the protozoan parasite *Trypanosoma cruzi*, found throughout the Americas in a variety of wild and domestic mammalian reservoirs, and it is usually transmitted by infected blood-sucking triatomine bugs. It is estimated that ~5.7 million people are currently infected with *T*. *cruzi* and that up to 120 million individuals living in endemic areas in Latin America are at risk of infection [2]. Chagas Disease remains the most important parasitic disease in the Western Hemisphere, with an estimated disease burden, as measured by disability-adjusted life-years, that is 7.5 times as great as that of malaria [2]. Increasing travel and immigration have also brought the risk of *T*. *cruzi* infection into non endemic countries [3]. Several efforts have successfully been undertaken to control transmission in Latin America, with a concomitant decrease in the number of acute vector-borne infections [4]. However, humans can also become infected with *T*. *cruzi* through the ingestion of tainted food and fluids, receipt of contaminated blood transfusion or organ transplantation, laboratory accidents, and from mother-to-child during pregnancy/delivery [1,4]. The diagnosis of Chagas disease is challenging because it is often asymptomatic in its acute phase and evolves into a chronic stage in which the disease presents diverse clinical forms [1]. In addition, and due to a major decline in parasitemia during the chronic phase, the detection of *T*. *cruzi* in blood samples by direct examination, hemoculture, or xenodiagnosis is difficult and time-consuming. Several PCR-based procedures have been reported that, although highly specific, present suboptimal sensitivity and require technological expertise and specialized expensive laboratory equipment [5]. In this framework, detection of anti-*T*. *cruzi* antibodies remains the most effective method for demonstrating direct exposure to the parasite [6]. At present, the most widely used serologic methods are indirect hemagglutination assay (IHA), indirect immuno-fluorescence assay (IIF), and enzyme-linked immunosorbent assay (ELISA) using total parasite homogenates or semipurified antigenic fractions [7]. Despite their satisfactory performance, these tests show variations in their reproducibility and reliability that can be attributed to poor standardization of the reagents or intrinsic variability of immune responses in patient populations [8–10]. In the absence of a single reference test showing 100% specificity and sensitivity, the current guidelines developed by the World Health Organization advise the use of two serologic tests for reaching a conclusive diagnosis. In the case of ambiguous or discordant results, diagnosis using a third technique should be conducted. In addition, there are other still unmet needs and gaps such as access to diagnostics in point-of-care sites for neglected populations [11,12], as well as development of much needed tests for early identification of congenital transmission; rapid assessment of drug treatment efficacy or prognostics tests for disease progression [10,13].

Recombinant DNA and peptide synthesis technologies historically allowed the production and one-step purification of large amounts of *T*. *cruzi* immunodominant antigens [14]. However, several studies showed that the use of single antigens in an assay did not confer the sensitivity required for a diagnostic test [14,15], which prompted the development of tests based on combinations of antigens[16,17], some of which were evaluated in multicenter trials and are commercially available [18–20]. Synthetic peptides are advantageous for diagnostic applications because they are: i) well defined (ease of quality control), ii) easily produced in large amounts, ii) highly pure and often cost-saving if compared to the production of natural or recombinant antigens *in vitro* [21]; and iv) also chemically stable (can be stored lyophilized or dessicated and tend to be stable for several years).

Short synthetic peptides spanning linear B-cell epitopes can also be used in serodiagnostic applications to increase specificity (that is, decrease the number of false positives) by replacing the use of whole protein antigens, therefore avoiding the display of unnecessary sequences that may lead to ‘false positive’ results. Specificity is a critical issue in serodiagnosis of Chagas Disease, where most reagents present cross-reactivity against other co-endemic parasites such as *Leishmania spp*. [18,21]. Peptide sensitivity, on the other hand can be increased using more densely presented immunoreactive epitopes (i.e. by creating a synthetic poly-epitopic molecule) or by combining multiple antigenic peptides in a single multiplex-assay [21–23]. A number of studies described the use of short peptides, containing either one or several epitopes for diagnosis of Chagas disease and other infectious diseases [23–34].

Recently, we have prioritized a number of candidate diagnostic targets from the genome of *T*. *cruzi* [35] and conducted a large-scale screening of parasite B-cell linear epitopes using high-density peptide microarrays [36]. This approach led to the identification of several hundred novel epitopes associated to chronic Chagas Disease, from which we selected 30 for further characterization. In this paper, we describe their diagnostic evaluation in ELISA format using a large panel of serum samples. In addition, and following an *in silico*-guided antigen combination strategy, we developed a proof-of-principle diagnostic kit based on these reactive peptides.

## Materials and methods

### Peptide selection

More than 2,000 candidate serodiagnostic peptides were previously identified by our group using a T. cruzi/Chagas HD peptide microarray [36]. To guide the selection of a subset of peptides for further serological characterization, a filtering strategy was conducted, as follows. First, peptides with serodiagnostic potential (high signal-to-noise ratio in the microarray experiments) were mapped to 187 distinct antigenic protein regions (stretches of adjacent peptides in a protein sequence). These antigenic regions may contain either a single B-cell linear epitope or, in some cases, a limited number of partially overlapping epitopes [37]. Next, antigenic regions were grouped into clusters of sequence-related peptides, in such a way that peptide sequences sharing stretches of 7 or more identical amino acids were put into the same cluster. We reasoned that peptides within a cluster may be both sequence and also likely antigenically related, whereas peptides from different clusters may likely represent the targets of different antibody specificities. From each cluster only a single antigenic region was kept (the one with highest microarray average seroreactivity). After this filter 95 unique antigenic regions were obtained (non-redundant, non-similar). From this set we selected 30 peptides from the top of the ranking for further characterization (the most reactive 15-mer from each antigenic region was selected). To minimize possible bias in our selection, the number of selected peptides from overrepresented sequences such as those from the mucin-associated surface protein (MASP) family [38] and from previously known antigens with mapped epitopes [24,39–43] was limited to 3 and 4, respectively. Sequence and features of our final set of synthetic peptides is summarized in Table 1.

**Table 1:** Sequence and features of synthetic peptides evaluated in this work. Bibliographic references are provided for the most relevant publication where the corresponding epitope has been mapped, or the protein has been validated as a human antigen.

Peptide	Protein	Description	Peptide sequence	Reference
**p1**	TcCLB.507071.20	mucin-associated surface protein (MASP)	LQVAGIKTTTATTGDS	This work
**p2**	TcCLB.506401.320	60S ribosomal protein L7a, antigenic protein	AKPAAKPAAKPAAKP	[35]
**p3**	TcCLB.506973.30	mucin-associated surface protein (MASP)	EKQQQSDEAQVQQHQ	This work
**p4**	TcCLB.511727.290	RNA-binding protein	PASKPAAKPAAKAPA	This work
**p5**	TcCLB.507083.109	hypothetical protein, conserved	WFEREVDGHDFIREH	This work
**p6**	TcCLB.507071.170	mucin TcMUCII	TTNAPSRLREIDGSL	[44]
**p7**	TcCLB.509793.50	hypothetical protein, conserved	KLGKSVGLTAALSPR	This work
**p8**	TcCLB.510101.430	40S ribosomal protein S21	GRDAPQARKQQGRNE	This work
**p9**	TcCLB.511679.10	mucin TcSMUGS	EGQYDAADVEAGDGP	This work
**p10**	TcCLB.506391.30	EF-hand protein 5	LMTREVDDTMADELR	[20]
**p11**	TcCLB.511529.80	kinetoplast DNA-associated protein	ALRVSPYSIFLQELA	This work
**p12**	TcCLB.511633.79	microtubule-associated protein	EEEEDVGPRHVDPDH	[45]
**p13**	TcCLB.506961.25	trans-sialidase	DSAKGKATGSSAGED	This work
**p14**	TcCLB.511287.120	40S ribosomal protein S2	RDPTDEHSDFLTMGS	This work
**p15**	TcCLB.506563.40	beta tubulin	PTGTYQGDSDLQLER	This work
**p16**	TcCLB.504159.10	hypothetical protein, antigenic protein n126	TSAPAAGGFGSATTT	[35]
**p17**	TcCLB.511633.79	microtubule-associated protein	PTTSARRLRTRTGPL	[45]
**p18**	TcCLB.510421.330	hypothetical protein, conserved	ILDRFLAAAMDKVFT	This work
**p19**	TcCLB.506989.190	heat shock protein 90, putative (LPG3)	PVDNDGDESSDKEDA	This work
**p20**	TcCLB.511633.79	microtubule-associated protein	VDPSAYKRALPLEEQ	[45]
**p21**	TcCLB.509157.120	hypothetical protein, conserved	SGAVPEGEEYPTEAE	[46]
**p22**	TcCLB.507071.100	mucin-associated surface protein (MASP)	SEREDDEENDEEEDG	This work
**p23**	TcCLB.511727.290	RNA-binding protein	GAAKAPAPKAAAPAP	This work
**p24**	TcCLB.511671.50	hypothetical protein, antigenic protein n96	AKPPAESPFKSVFGA	[35]
**pc1**	TcCLB.508831.140	B13 / Ag2 / CA-2 / PEP2	APFGQAAAGDKPSPF	[41]
**pc2**	TcCLB.509149.40	Ribo L19	AAAPAKAAAAPAKAA	[24]
**pc3**	TcCLB.505975.20	TcD / Ag13	EPKSAEPKPAEPKSA	[45]
**pc4**	X57235	Trans-sialidase (SAPA)	TPADSSAHSTPSTPA	[43]

Peptides in Table 1 were synthesized and used in ELISA assays as described below (see also Results) to screen for reactivity against Chagas positive and negative (control) samples. Once we obtained a first matrix of reactivity of peptides vs individual serum samples, we applied the EpiSelect algorithm to guide the selection of sets of peptides for the formulation of multiepitope assays. Implementation of the algorithm has been described [47], but briefly the algorithm aims to find the smallest selection of peptides (epitopes) that in concert maximizes the coverage (reactivity) against a given set of subjects. The input to the algorithm was the matrix of peptide reactivity values determined by ELISA, encoded as z-scores defined as the number of standard deviations above background. Positive peptides were defined using a z-score threshold of 3.

### Synthetic peptides and BSA conjugation

Synthetic peptides were purchased from Schafer-N (Copenhagen, Denmark). Peptides were synthesized using standard FMOC chemistry, purified by HPLC (> 90% purity) and characterized by mass spectroscopy. A C-terminal cysteine residue was included in all peptides for conjugation to maleimide-activated BSA. An additional amino acid residue (leucine) was added at the N-terminus of peptide p1, to avoid the partial deamination associated with an N-terminal glutamine [48]. Lyophilized peptides were resuspended in sterile-filtered water (Sigma Product w3500), and conjugated to maleimide-activated BSA (mBSA, Sigma-Aldrich Product B7542) according to the manufacturer’s protocol, using a molar ratio of 35:1 peptide to mBSA [49]. Peptide-mBSA conjugates were stored in 50% glycerol at -20°C until use. Peptides that failed to solubilize under these conditions were discarded for the analysis.

### Human serum samples, samples size and error estimation

Human serum samples from *T*. *cruzi*-infected patients used in this study were obtained from the Laboratorio de Enfermedad de Chagas, Hospital de Niños "Dr. Ricardo Gutierrez" (HNRG, Buenos Aires, Argentina) (n = 80). Human serum samples from patients with American Tegumentary Leishmaniasis (ATL) used in this study were obtained from the Instituto de Patología Experimental, Universidad Nacional de Salta (IPE, Salta, Argentina) (n = 19). All procedures were approved by the research and teaching committee and the bioethics committee of both institutions, and followed the Declaration of Helsinki Principles. Written informed consent was obtained from all individuals (or from their legal representatives), and all samples were decoded and de-identified before they were provided for research purposes. Chagasic patients were in the asympomatic chronic stage of the disease without cardiac or gastrointestinal compromise (age range: 11 to 51 years old, median age: 20). Serum samples were collected from clotted blood obtained by venipuncture and analyzed for *T*. *cruzi*-specific antibodies with the following commercially available kits: ELISA using total parasite homogenate (Wiener lab, Argentina) and IHA (Polychaco, Buenos Aires, Argentina). ATL patients were diagnosed using a combination of techniques: direct observation of parasites (amastigotes) on smears of dermal scrapings; a test of delayed-type hypersensitivity (Montenegro skin test); and a clinical assessment (see [50]). The negative panel was composed of samples from healthy, non-infected individuals that gave negative results in the aforementioned tests, and were obtained either from the blood bank “Fundación Hemocentro Buenos Aires” (FHBA Buenos Aires, Argentina) (n = 82) or from IPE (n = 18). Samples from FHBA were also negative for HIV, Hepatitis B, Hepatitis C, HTLV I and II, *Treponema pallidum* (syphilis) and for Brucelosis (Huddlesson test).

To calculate the minimum sample size required to estimate sensitivity or specificity for a specified interval of confidence and precision under a normal approximation, we used the following formula:

$$n=\frac{Z^{2}\;\hat{P}\;\left(1-\hat{P}\right)}{d^{2}}$$

Where Z is the z-score from a standard normal distribution (e.g. 1.96 for a 95% confidence interval), $\hat{P}$ is the pre-determined (guess) value of sensitivity (or specificity) based on previous experience/judgment, and ***d*** is the required precision [51]. Therefore, for **Z** = 1,96 (95% CI), $\hat{P}$ = 0.99, and ***d*** = 0.05 (5% error), the estimated sample size is 73. Therefore 73 is the minimum number of Chagas positive samples (to estimate sensitivity) and Chagas negative samples (to estimate specificity).

### ELISA assays and statistical analysis

Microplates containing 96 or 384-wells (Thermo Scientific ImmunoPlates, MaxiSorp) were coated overnight at 4°C with 100 ng/well of peptide-mBSA or with different peptide mixtures (80 ng/well of each one) in PBS pH 7.4. Blank signal was determined using mBSA-coated wells. After 4 washings with TBS-T (50 mM Tris-HCl (pH 7.6), 150 mM NaCl, 0.05% (v/v) Tween20), the plates were blocked for 1 h at room temperature with 100 μl/well of assay buffer (3% (w/v) skimmed milk in TBS-T). The plates were washed and incubated for 1 h with human sera diluted as indicated (1:100 or 1:10) in assay buffer at room temperature. Optimization of the assay conditions was performed by a checkerboard titration analysis using 10 ng or 80 ng of peptide-mBSA, and different dilutions of secondary antibody (peroxidase-conjugated goat anti-human IgG antibodies (Sigma-Aldrich, St Louis, MO) (1:5,000; 1:10,000; 1:20,000 and 1:80,000). After washings, 100 μl of secondary antibody diluted as indicated (1:10,000 for assays using a single peptide per well, or 1:80,000 for multiepitope assays) in assay buffer were added to each well and incubated for 1 h at room temperature. Following additional washings with TBS-T, the reaction was developed with tetramethylbenzidine for 15 min (TMB, Sigma-Aldrich, St Louis, MO) and stopped by addition of 0.2 M sulphuric acid. Absorbance values were measured at 450 nm in a microplate absorbance reader (FilterMax F5 Multimode, Molecular Devices, Sunnyvale, CA, USA). All serum samples were tested in duplicate. Values were averaged and blank-corrected.

### Data analysis

The same 16 serum samples from healthy blood donors were tested in each ELISA plate. The cut-off value was determined for each peptide and for each plate using the mean of the control blood donor samples plus 3 SD (the cut-off was set accounting for multiple-hypothesis testing). For each peptide or peptide mixture, standardized reactivity scores (z-scores) and the diagnostic analytical characteristics of sensitivity, specificity and AUC (Area under the ROC–Receiver Operating Characteristic–curve, as a performance metric) were calculated. Reagent sensitivity was calculated as the number of positive subjects (i.e. infected patients samples that were reactive against a particular peptide) over the total number of infected subjects tested; specificity was calculated as the number of negative subjects (non-infected control subjects that were seronegative against a particular peptide) over the total number of non-infected control subjects tested and AUC was calculated using the from the z-scores of infected subjects and non-infected subjects. For receiver operating characteristic (ROC) analyses [52], the results were expressed as the percentage of reactivity of the mean absorbance at 450 nm of the positive reference control serum included in each assay run. The Mann-Whitney test and ROC analysis were performed using the GraphPad Prism software (version 6 for OSX; San Diego, CA, USA) or ROCR R package [53].

## Results

### Diagnostic performance of selected peptides in ELISA format

**Fig 1:**
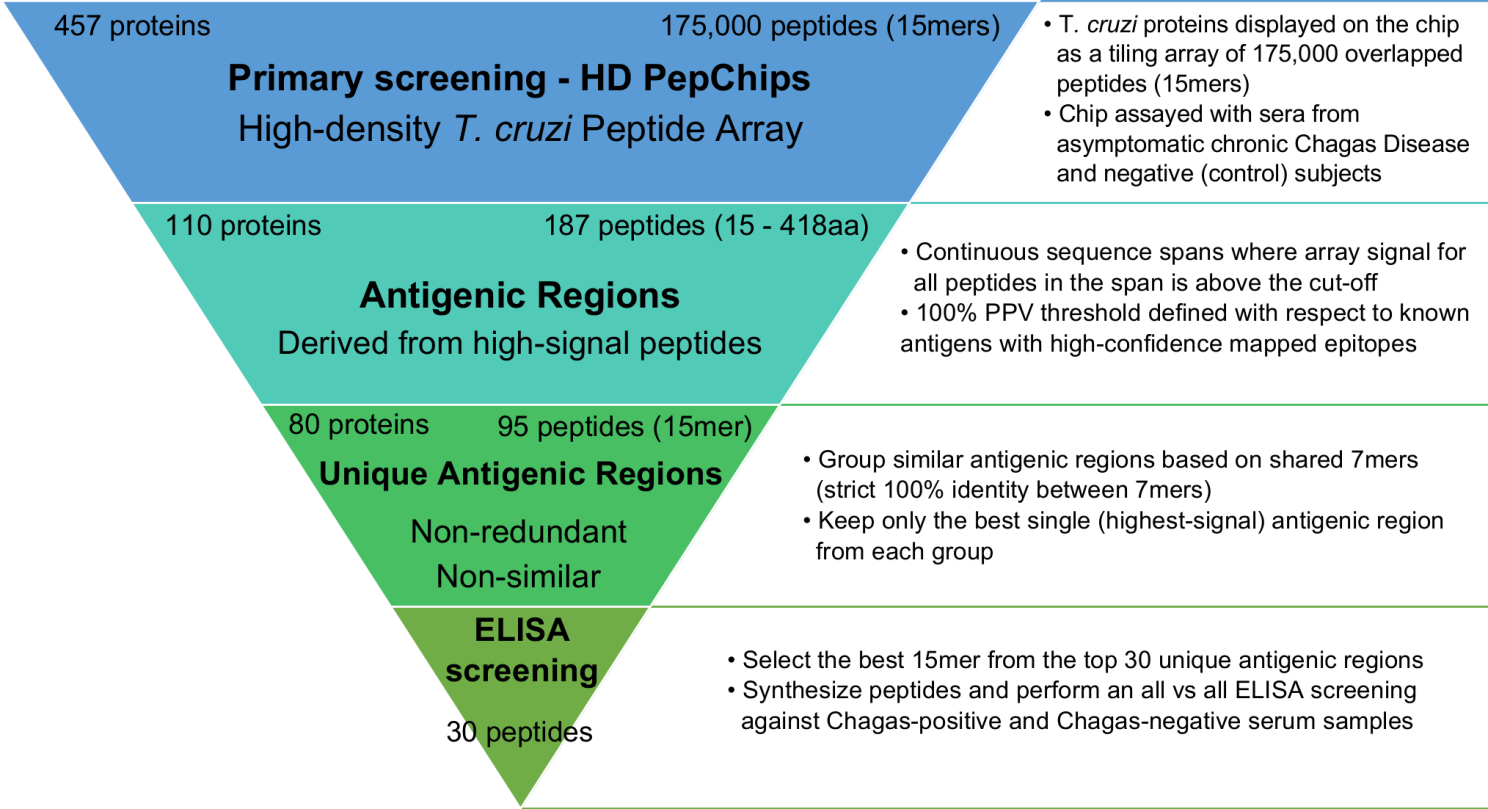
Flowchart showing the strategy for peptide selection.

Based on our previous screening of serodiagnostic peptides for Chagas Disease using HD peptide microarrays [36], 30 peptides were selected for further serological characterization and downstream validation. The strategy for selection of these peptides is outlined in Fig 1 (see also Methods), and essentially was guided to select a non-redundant set of peptides showing the highest antibody-binding signal in any array. After removing 3 peptides that showed solubility problems, the remaining 27 peptides were coupled to a carrier protein (mBSA) and assayed in ELISA format against a sera panel of 62 chronically infected Chagasic patients and 16 healthy controls. Initially, all human sera were tested at 1:100 dilutions. The panel of peptides included 16 peptides corresponding to previously uncharacterized *T*. *cruzi* proteins (novel antigens) that emerged during our screening [36], 7 peptides representing novel epitopes in previously characterized B-cell antigens and 4 peptides corresponding to previously known linear B-cell epitopes, which were used as positive controls (see Table 1 and S1 Fig). We also included in our panel an additional peptide (p17) as an internal negative control. Although belonging to a validated *T*. *cruzi* antigen [54], this peptide was derived from a protein region that showed consistently very low signal in all microarray replicates.

Diagnostic sensitivity, specificity and AUC values for each peptide are shown in Table 2 (complete data available in S1 Table). The diversity of reactivities in the collection of sera samples when assayed against individual peptides is also evident when visualizing the data in the form of a heatmap plot (available in S2 Fig). As shown, promising diagnostic performances were observed for most of the assayed peptides. Sensitivity values ranged from 30–92% (>50% in 22 out of 27), and specificity values were extremely high, which is consistent with our screening strategy [36]. In this context, it is worth noting that sensitivity values of all individual *T*. *cruzi* antigens described so far and proposed and/or included in serodiagnostic tests ranged from 80–99% [14].

**Table 2:** Diagnostic performance of selected peptides in ELISA format. Peptides are sorted per decreasing sensitivity.

Peptide	Avg signal (pepchips)	Sensitivity (n = 62)	Specificity	ROC AUC
**pc1**	46,23	91,98	98.36 (n = 61)	0,99
**pc2**	35,22	90,31	96.72 (n = 61)	0,98
**p7**	17,19	84,06	96.72 (n = 61)	0,94
**p11**	13,43	84,06	95.1 (n = 61)	0,96
**p16**	10,89	83,85	98.36 (n = 61)	0,94
**p1**	64,57	80,94	98.36 (n = 61)	0,94
**p19**	7,39	80,94	95.1 (n = 61)	0,95
**p5**	26,15	80,63	98.36 (n = 61)	0,96
**pc3**	13,66	78,96	100 (n = 61)	0,97
**pc4**	0,08	77,08	95.1 (n = 61)	0,94
**p12**	11,73	76,15	96.72 (n = 61)	0,94
**p24**	4,45	74,17	100 (n = 61)	0,95
**p6**	23,56	69,06	98.36 (n = 61)	0,9
**p21**	6,87	65,94	95.1 (n = 61)	0,88
**p18**	7,63	64,79	96.72 (n = 61)	0,91
**p13**	11,53	60,42	96.72 (n = 61)	0,88
**p15**	11,1	59,58	100 (n = 16)	0,81
**p2**	50,61	57,81	100 (n = 16)	0,88
**p4**	26,52	56,04	100 (n = 16)	0,88
**p10**	13,78	54,79	100 (n = 16)	0,88
**p3**	31,06	53,65	100 (n = 16)	0,8
**p20**	4,3	50,1	100 (n = 16)	0,83
**p8**	16,7	45,83	93,8 (n = 16)	0,76
**p14**	11,22	45,73	93,8 (n = 16)	0,76
**p22**	6,65	32,4	100 (n = 16)	0,72
**p23**	5,85	30,42	100 (n = 16)	0,77
**p9**	15,68	29,06	93,8 (n = 16)	0,73
**p17**	0	3,23	100 (n = 16)	ND

Overall, and as previously reported for the TSSA antigen [37], a strong correlation between assays in the standard ELISA format and in microarray format was observed for each peptide (Table 1), thus providing additional validation and support for the use of HD-peptide arrays for discovery of new serology-based biomarkers.

### Extended evaluation of diagnostic specificities of top-ranked peptides

We further evaluated the diagnostic specificity of the 16 best performing peptides (see Table 1) by using an extended panel of 61 control sera obtained from healthy subjects (Chagas-negative samples). As before, individual peptides coupled to mBSA were assayed in ELISA format. Diagnostic specificities and ROC-AUC were re-calculated for each peptide (top entries in Table 2). The average specificity was 97.23% and in all cases specificities > 95% were observed. Notably, most of the positive responses observed in this expanded set of Chagas-negative samples correspond to only 3 of the 61 sera samples tested. These samples (also negative for the highly-sensitive trans-sialidase inhibition assay [55]) were highly reactive against more than half of the peptides (12, 11 and 9 peptides each, see S1 Table in the ‘Additional negative sera’ section), suggesting a broad and yet-to-be explained cross-recognition towards *T*. *cruzi*-derived sequences. If these Chagas-negative serum samples were removed, specificity values of our peptides would increase up to an average 98.5%.

### A novel multiepitope diagnostic method for Chagas Disease

**Fig 2:**
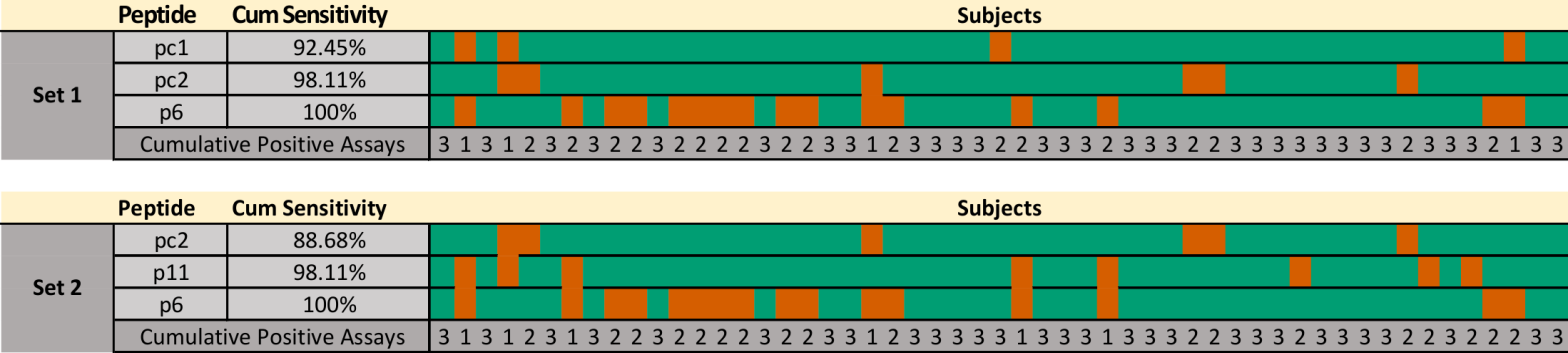
Reactivity pattern of example optimal peptide subsets. Two peptide combinations were created using the EpiSelect algorithm to achieve a theoretical 100% sensitivity, based on data from the individual assays in S1 Table (53 Chagas positive samples, 27 peptides). The figure shows positive (green) and negative (red) results for each combination of peptide and subject. The cumulative sensitivity is also indicated. For each set, the bottom row displays the cumulative number of positive results for each subject.

Based on the results described above, we undertook an *in silico*-guided approach to design a multiplex assay with improved diagnostic performance. Using ELISA data from individual peptides, we applied the EpiSelect algorithm [47] (see Methods) to identify several optimal (minimal) virtual peptide sets that in concert provided maximal coverage of the analyzed subjects. This analysis was performed after removing data from the 9 serum samples that were previously used in microarray experiments, to avoid optimistically biased results. The analysis performed on the tested peptides and 53 Chagas-positive subjects showed that 3 peptides were enough to reach a theoretical sensitivity of 100% (Fig 2). Data used for this analysis is available in S1 Table. The optimal set was composed by peptides {pc1, pc2, and p6}, resulting in an average of 2.51 reactive peptides per subject, closely followed by the peptide set {pc2, p11, and p6} with an average of 2.43 reactive peptides per subject. The reactivity patterns for these sets are shown in Fig 2 and S1 Table. Interestingly, at least 1 of the 3 novel peptides p6 (as in Fig 2), p2 or p8 (alternatives) would be required to achieve a sensitivity of 100% with a 100% specificity (see also S1 Table).

Other peptides such as p5, p7, p11, p12, p16, p19 and p24 also displayed excellent diagnostic characteristics, with individual high sensitivity (> 70%) and specificity (up to 95%). Hence, these peptides can be eventually incorporated into the multiplex design to increase its robustness (for example, to increase the number of reactive peptides per subject).

Based on these analyses, we prepared and tested a number of multi-epitope peptide combinations in ELISA format against an extended panel of sera from chagasic (positive) and healthy (negative) subjects. One such combination {pc1, pc2, pc3, p6, p13}, was tested against 22 positive and 24 negative serum samples and gave a diagnostic sensitivity of 72.7% and a specificity of 91.7%. Following the same methodology (S1 Table), we tested a slightly different formulation of peptides (pc1, pc2, p6, p7 and p24) against an increased number of sera samples (53 Chagas-positive and 31 Chagas-negative) obtaining an improved performance, with a sensitivity of 92.45% and a specificity of 93.55%.

**Fig 3:**
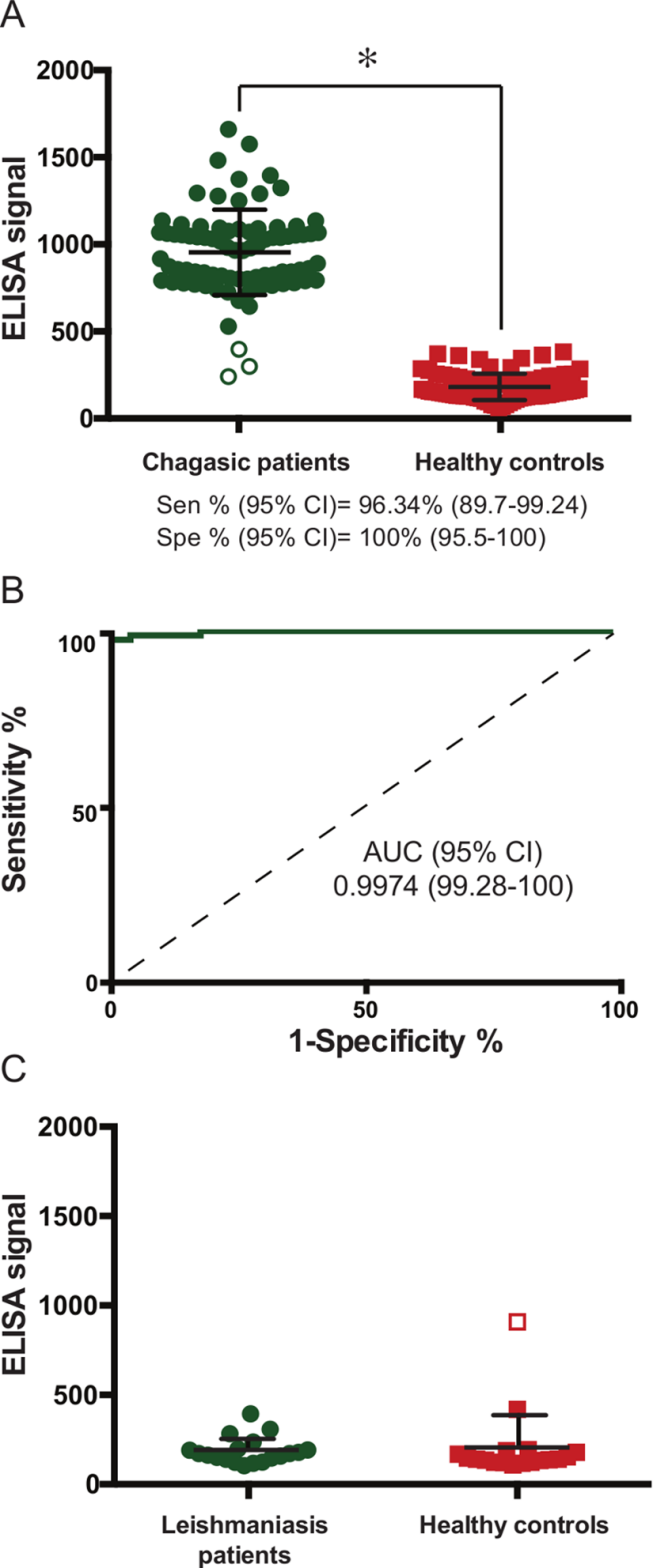
Diagnostic performance of the final multiepitope formulation. **A)** Scatter plot showing the distribution of ELISA raw signal obtained from sera samples of Chagas-positive (green circles) and healthy subjects (red squares). Statistical significance (*) p<0.0001, Mann-Whitney test. Green empty circles in the left scatter plot represent those within 3 standard deviations of the average signal of healthy controls. **B)** Receiver Operating Characteristic curve and statistics for the analysis. **C)** Scatter plot showing the distribution of ELISA raw signal obtained from Leishmaniasis-positive (green cirles) and Leishmaniasis-negative subjects from the same co-endemic region (red squares). Empty circles represent data points that fall at >3 SD from their respective distributions.

Finally, with the aim of obtaining a peptide combination with enhanced robustness, we re-analyzed the reactivity profile of each individual serum sample (S1 Table) against our panel of peptides, and identified a few Chagas positive subjects that gave low or even negative reactivity to many peptides. From this analysis, we identified peptides that would theoretically maximize the sensitivity of the multiplex assay, despite not showing the best possible coverage of our subject (sera) collection. Thus, we arrived at a high performance multi-epitope formulation of seven peptides {pc1, pc2, pc3, p6, p7, p13, and p24}. To validate this final formulation, we increased the amount of coated peptide to 80 ng of each peptide per well and the serum concentration to 1:10. After these modifications, the performance of this formulation, when tested against 82 Chagas-positive and 80 Chagas-negative sera samples gave a sensitivity of 96.34% and a specificity of 100%, with an AUC value of 0.9974 (Fig 3).

We have also assessed the performance of this multiepitope formulation against a panel of 19 sera from subjects with positive diagnosis for American Tegumentary Leishmaniasis (see Methods), and another 18 negative (control sera) from the same endemic region. Only a single (negative) subject gave a positive response in our multiepitope assay (Fig 3C). Except for this case, the observed absorbance in the ELISA assays was nil. The specificity of the multiepitope formulation for this panel was 97.30%, with an overall specificity (considering all negative samples from all panels) of 99.15%. Table 3 summarizes the performance of this combination of peptides. This therefore represents a highly promising novel multiepitope formulation for the diagnosis of Chagas Disease.

**Table 3:** Summary of performance of the final multiepitope combination.

Multi-epitope combination	pc1, pc2, pc3 + p6, p7, p13, p24
**Sensitivity (True Positive Rate)**	96.34%
**Specificity (True Negative Rate)**	99.15%
**Positive Predictive Value**	98.75%
**Negative Predictive Value**	97.47%

## Discussion

Serological diagnostics methods for infectious diseases have usually evolved from first-generation lysate-based reagents. Through time, more defined formulations of diagnostic reagents have followed. Second-generation diagnostic kits based on purified antigenic fractions or third-generation kits based on recombinant proteins are now in widespread use. To develop new diagnostic tools that are simple and have few manipulation steps, one of the central aspects that currently limits the suitability of diagnostic kits is the need to produce, prepare and purify the antigens, along with the corresponding quality control. Short synthetic peptides can be produced cheaply in large quantities, and are chemically stable and amenable for long-term storage. Synthetic peptides have been already tested in a wide range of diagnostic applications and proved valuable for diagnosis of viral, bacterial, parasitic and autoimmune diseases [21,30–34]. Therefore, fourth-generation diagnostic kits based on well-defined peptidic antigens are now within reach.

Here we present a next-generation diagnostic formulation for Chagas Disease based on short peptides. Significant efforts have been invested by various groups over time to identify and test antigenic peptides for serodiagnosis of Chagas Disease, some of which displayed promising analytical characteristics. For example, peptides Ag2/B13/Pep2, TcD/Ag13, TcE and TcLo1.2, have been combined to create a multi-epitope recombinant neo-protein of excellent performance [24], and peptides from the cytoplasmic repetitive antigen (CRA)/Ag30 and flagellar repetitive antigen (FRA)/Ag1 [54] have been recently shown to present good specificity and sensitivity [56].

The advent of novel high-throughput approaches spawned by the post-genomic era is starting to impact on the discovery of new biomarkers and the development of diagnostic tools for a number of important pathogens [10]. We have recently showed the utility of a fast approach to screen for new *T*. *cruzi* antigens that is based on high-density peptide microarrays [36]. The advantage of this platform is that it allows to identify antigens and at the same time obtain a fine mapping of their linear epitopes. Using this strategy we have identified and mapped the epitopes of >90 novel *T. Cruzi* antigens [36].

As a followup of this first screening for peptidic antigens, we provide here an extensive serological characterization of 27 peptides, 18 of which represent novel epitopes that were mapped using our strategy, or represent recently discovered antigens but for which no fine epitope mapping was yet available (see Table 1). For example, even though the trans-sialidase/SAPA antigen (accession number X57235, TcCLB.509495.30 is the most similar genome locus tag) has been known for quite some time, peptide p13 (also annotated as ‘trans-sialidase’) is not derived from the originally described antigen, but from another member of the superfamily (TcCLB.506961.25) with only 29% identity to the original trans-sialidase/SAPA. Therefore, p13 is a new/novel antigen and epitope that bear no resemblance to any of the mapped epitopes already described [43,57]. Similarly, even though the proteins encoded by the genes TcCLB.511633.79 (microtubule-associated protein), or TcCLB.506391.30 (EF-hand protein 5) were already described and used as antigens [20,46], this is the first time that their fine mapped epitopes are tested for diagnostic purposes. Other peptides such as p16, p7, p11 and p19 are part of proteins that have been identified as potential antigens [35] but with no other serological evidence before our microarray experiments. Peptide p1, on the other hand, was derived from a member of the Mucin-Associated Surface Protein (MASP) family [38], which is a large family of genes which were shown recently to be the target of the adaptive immune response in an animal model of infection [58]. The MASP protein encoded by gene TcCLB.507071.20 was selected from the genome, as part of an effort to obtain a detailed characterization of the antigenicity and epitopes of this gene family in human infections [59]. Peptide p6 contains a slightly different version of the sequence TTRAPSRLREID, which has been identified as the major and conserved linear B-cell epitope included within the otherwise highly polymorphic TcMUCII family of *T*. *cruzi* proteins [44,60]. Whereas peptide p2 is a novel epitope from a putative 60S ribosomal protein L7a, that we have also previously identified as a potential antigen [35].

Using a panel of Chagas-positive and negative (control) samples, we performed a thorough serological characterization of the selected peptides. This allowed us to obtain a relatively large matrix of ELISA responses for all peptides against individual serum samples. This led us to identify a number of peptides with promising diagnostic potential, such as peptides p1, p7, p11, p16 and p19, which presented sensitivities above 80%, with no false positive responses in the first evaluation using a small panel of 16 sera, and only a few false positive responses (with specificities from 96.5% to 100%) in a second evaluation using a larger panel of sera. These sensitivities are similar to those originally reported in the first characterizations of validated serodiagnostic antigens such as TcD (95% for chronic subjects [61]) and SAPA (10% for chronic subjects, 90% for acute infection [62]), which were later improved when developed into a multiantigen diagnostic reagent (e.g. the Chagatest kit of Wiener Labs that includes these antigens claims a sensitivity of 98.8%[63]). Hence, even if some peptides displayed sensitivities that were not very high when assessed singly, they were high enough as to keep them under consideration for development of an assay based on combinations of peptides.

The matrix of ELISA responses was then used to guide the rational formulation of a multiepitope diagnostic reagent using a well-defined algorithm for the inclusion of peptides. The first combinations tested did not achieve a significantly high performance, even if the theoretical prediction (Fig 2) would suggest otherwise. One reason for this is that even though the input to the EpiSelect algorithm included the level of response of each subject against each peptide (represented as the number of standard deviations above negative controls), the effect of combining peptides produced a higher background signal that was not predicted by the algorithm. Another reason was the inclusion in our panel of Chagas-positive sera of several samples with moderately low antibody titers overall (see for example the 9 sera grouped in the bottom branch in S2 Fig). Despite these pitfalls, the detailed data present in this matrix was pivotal in identifying peptides for inclusion in the final multiepitope formulation. The rationale for inclusion of peptides was the ability of a given peptide (as observed in the matrix) to potentially overcome a negative response for a given serum sample. For example, peptides p6 and p2, followed by p11 represented an optimal complement of the two best performing peptides, pc1 (from the antigenic repeat of the CA-2/B13 antigen Ag2) and pc2 (the serodiagnostic epitope TcE) for diagnosis. Also, peptide p13 when combined with peptides pc1 and pc2 was one of the few peptides that provided relatively high signal in the ELISA assay against the group of sera with relatively low overall responses. The fact that we could consistently increase the performance of each combination upon following this rationale shows the usefulness of this approach.

Interestingly, all peptides in the final multiepitope formulation are highly conserved (see S1 Text). A sequence similarity search across available complete genomes (e.g. those from the CL-Brener [64] and Sylvio X10 [65] strains using BLASTP) or from draft assemblies (Tula cl2, Esmeraldo cl3, Dm28c or JRcl4 in the TriTrypDB resource [66], release 30 from February 2017, using TBLASTN) shows that all peptides are highly conserved across strains representing different evolutionary lineages of the parasite (TcI, TcII, TcV, TcVI).

The observed diagnostic performances for all peptides and peptide combinations tested were very promising, particularly considering that all assays were based on short synthetic peptides. Our final best performing multi-epitope combination was based on a combination of seven antigenic peptides. With an equimolar mixing of peptides, we attained a very high (>96%) level of sensitivity and specificity. These are highly promising values for a first optimization attempt; the final ELISA assay/formulation could be indeed further improved using different blocking reagents, coupled detection system and, most importantly, by adjusting the relative concentration of different peptides in the final mixture.

Analysis of potential cross-reactivity with other co-endemic diseases and pathogens is essential to validate any diagnostic reagent. In the case of Chagas Disease, cross-reactivity against infections with Leishmania species is a particular concern [67]. We have included a panel of serum samples from confirmed cases of tegumentary leishmaniasis from the northern province of Salta, Argentina to assess the performance of our formulation. This also gave us the opportunity to improve the assessment of specificity by analizing a paired set of negative (control) samples (chagas-negative and leishmaniasis-negative) from the same endemic region. From a set of 37 of these samples which were negative for Chagas Disease, only one gave a positive cross-reactive response (Fig 3). Although this highlights the need to perform a more extensive characterization of this cross-reactive sample (e.g. against our complete panel of peptides), and eventually revise the combination of peptides in our formulation, the current multiepitope assay has a sufficiently high specificity at this stage (99.15%), comparable to other commercially available kits [63] that can certainly be improved by optimization of the assay or by replacing of cross-reactive peptides.

Besides the obvious attention to the diagnostic performance of the identified peptides, these results serve to validate the use of high-density peptide microarrays as a fast screening platform. The fact that all selected peptides gave positive responses against several Chagas-positive subjects show that this technology can be trusted to rapidly identify and map epitopes of complex pathogens. It is also worth mentioning here that there are about a hundred additional antigenic regions within the signal range observed in the peptide microarray screening from which these peptides were identified [36] and that await further serological characterization. This observation, together with the fact that the microarray screening only covered ~3% of the parasite proteome, show that there is still a large repertoire of Chagas-specific antibody specificities that remain serologically unexplored.

The results presented herein hence provide a novel, robust multi-epitope formulation as a basis for the development of improved peptide-based serodiagnostics for Chagas Disease. In contrast with chimeric DNA constructs that encode multiepitope recombinant proteins, the fact that this diagnostic reagent is based on the combination of short peptides that can be synthesized separately and easily formulated in a mix-and-match approach, means that it can be improved successively over time with only a reasonable effort.

## Supporting information

S1 Fig Antibody binding profiles of antigens showing the location of selected peptides. The antibody binding profiles of antigens were derived from previously published data [36](ArrayExpress accession number E-MTAB-3008). Briefly, high-density peptide microarray slides were assayed with purified immunoglobulins from healthy subjects (four pools of samples labeled A-D, shown as dashed lines) or Chagas positive subjects (four pools of samples labeled A-D, shown as solid lines). Antibody binding profiles were reconstructed for each of the selected antigens as described previously. Each plot in the figure shows the normalized and smoothed signal profile for a single antigen (one per page). A different scale may be used in each plot to best accommodate all peaks. The location of each the selected peptides used in this study is shown in context with other antigenic regions in each antigen. File: S1 Fig.
(PDF)

S2 Fig Heatmap plot showing the pattern of reactivity of peptides against a panel of positive sera. Heatmap display of ELISA reactivity of each of the 27 peptides tested against a panel of 62 positive sera samples. For the heatmap display the reactivity values (in the form of z-scores above background) were transformed for clarity using a sigmoid function centered around 3. Peptides and subjects were clustered using a hierarchical clustering algorithm (R, hclust). A group of subjects showing moderately low ELISA reactivity across peptides has been highlighted (see main text). File: S2 Fig.
(PDF)

S3 Fig STARD flow diagram for studies reporting diagnostic accuracy. (PDF)

S1 Table Detailed results of ELISA assays. The spreadsheet workbook file contains a number of worksheets with results from different ELISA assays: 1) all vs all ELISA results (N = negative; P = positive) for each of the 27 peptides against 62 sera samples from chronically infected (Chagas-positive) patients and 16 negative controls (healthy subject); 2) all vs all (z-scores) contains the input matrix for the EpiSelect algorithm; 3) additional negative sera, ELISA results for the best performing 16 peptides against an additional panel of 61 negative sera samples; 4) Formulation 1, ELISA results for the combination of peptides {pc1, pc2, pc3, p6, p13}; 5) Formulation 2, ELISA results for the combination of peptides {pc1, pc2, p6, p7, p24}; 5) Final formulation, ELISA results for the combination of peptides {pc1, pc2, pc3, p6, p7, p13, p24}. File: S1 Table.
(XLSX)

S2 Table STARD checklist for studies reporting diagnostic accuracy. (PDF)

S1 Text Conservation of peptides and epitopes across evolutionary Trypanosoma cruzi evolutionary lineages. This supporting file contains information on the conservation of the selected epitopes. We have tried to compile information from complete *T. cruzi* genomes from different evolutionary lineages (Discrete Typing Units, DTUs). For each peptide (naming/numbering follows Table 1), we provide a small multiple sequence alignment showing presence and conservation of the peptide in other strains/isolates. In the case of hybrid lineages more than one representative sequence may have been included in the alignment. File: S1 Text.
(TXT)

## References

[1] Rassi A Jr, Rassi A, Marin-Neto JA (2010) Chagas disease. Lancet 375: 1388–1402. Available: 10.1016/S0140-6736(10)60061-X. 20399979

[2] Bern C (2015) Chagas’ Disease. N Engl J Med 373: 456–466. Available: http://www.ncbi.nlm.nih.gov/pubmed/26222561. Accessed 17 July 2017. 10.1056/NEJMra1410150 26222561

[3] Gascon J, Bern C, Pinazo M-J (2010) Chagas disease in Spain, the United States and other non-endemic countries. Acta Trop 115: 22–27. Available: http://linkinghub.elsevier.com/retrieve/pii/S0001706X09001995. Accessed 17 April 2015. 10.1016/j.actatropica.2009.07.019 19646412

[4] Muñoz-Calderón A, Díaz-Bello Z, Valladares B, Noya O, López MC, et al (2013) Oral transmission of Chagas disease: typing of Trypanosoma cruzi from five outbreaks occurred in Venezuela shows multiclonal and common infections in patients, vectors and reservoirs. Infect Genet Evol 17: 113–122. Available: http://www.ncbi.nlm.nih.gov/pubmed/23567816. Accessed 26 May 2015. 10.1016/j.meegid.2013.03.036 23567816

[5] Brasil PEAA, De Castro L, Hasslocher-Moreno AM, Sangenis LHC, Braga JU (2010) ELISA versus PCR for diagnosis of chronic Chagas disease: systematic review and meta-analysis. BMC Infect Dis 10: 337 Available: http://www.pubmedcentral.nih.gov/articlerender.fcgi?artid=3004908&tool=pmcentrez&rendertype=abstract. Accessed 18 March 2015. 10.1186/1471-2334-10-337 21108793 PMC3004908

[6] Gomes YM, Lorena VMB, Luquetti AO (2009) Diagnosis of Chagas disease: what has been achieved? What remains to be done with regard to diagnosis and follow up studies? Mem Inst Oswaldo Cruz 104 Suppl: 115–121. Available: http://www.ncbi.nlm.nih.gov/pubmed/19753466. Accessed 17 April 2015. 19753466 10.1590/s0074-02762009000900017

[7] Balouz V, Agüero F, Buscaglia CA (2017) Chagas Disease Diagnostic Applications: present knowledge and future steps. Advances in parasitology. Vol. 97 pp. 1–45. Available: http://www.ncbi.nlm.nih.gov/pubmed/28325368. Accessed 16 August 2017. 10.1016/bs.apar.2016.10.001 28325368 PMC5363286

[8] Afonso AM, Ebell MH, Tarleton RL (2012) A systematic review of high quality diagnostic tests for Chagas disease. PLoS Negl Trop Dis 6: e1881 Available: http://www.pubmedcentral.nih.gov/articlerender.fcgi?artid=3493394&tool=pmcentrez&rendertype=abstract. Accessed 26 May 2015. 10.1371/journal.pntd.0001881 23145201 PMC3493394

[9] Moure Z, Angheben A, Molina I, Gobbi F, Espasa M, et al (2016) Serodiscordance in chronic Chagas disease diagnosis: a real problem in non-endemic countries. Clin Microbiol Infect. 10.1016/j.cmi.2016.06.001 27317907

[10] Buscaglia CA, Kissinger JC, Agüero F (2015) Neglected Tropical Diseases in the Post-Genomic Era. Trends Genet 31: 539–555. Available: http://www.cell.com/article/S0168952515001134/fulltext. Accessed 7 October 2015. 10.1016/j.tig.2015.06.002 26450337

[11] Peeling RW (2015) Diagnostics in a digital age: an opportunity to strengthen health systems and improve health outcomes. Int Health 7: 384–389. Available: http://www.ncbi.nlm.nih.gov/pubmed/26553825. Accessed 29 July 2016. 10.1093/inthealth/ihv062 26553825 PMC7108565

[12] Peeling R (2015) Bringing diagnostics to developing countries: an interview with Rosanna Peeling. Expert Rev Mol Diagn 15: 1107–1110. Available: http://www.ncbi.nlm.nih.gov/pubmed/26312948. Accessed 29 July 2016. 10.1586/14737159.2015.1081802 26312948

[13] Pecoul B, Batista C, Stobbaerts E, Ribeiro I, Vilasanjuan R, et al (2016) The BENEFIT Trial: Where Do We Go from Here? PLoS Negl Trop Dis 10: e0004343 Available: http://dx.plos.org/10.1371/journal.pntd.0004343. Accessed 29 July 2016. 10.1371/journal.pntd.0004343 26913759 PMC4767872

[14] da Silveira JF, Umezawa ES, Luquetti AO (2001) Chagas disease: recombinant Trypanosoma cruzi antigens for serological diagnosis. Trends Parasitol 17: 286–291. Available: http://www.ncbi.nlm.nih.gov/pubmed/11378036. Accessed 20 March 2015. 11378036 10.1016/s1471-4922(01)01897-9

[15] Levin MJ, Franco da Silveira J, Frasch AC, Camargo ME, Lafon S, et al (1991) Recombinant Trypanosoma cruzi antigens and Chagas’ disease diagnosis: analysis of a workshop. FEMS Microbiol Immunol 4: 11–19. Available: http://www.ncbi.nlm.nih.gov/pubmed/1815706. Accessed 1 August 2017. 1815706 10.1111/j.1574-6968.1991.tb04965.x

[16] Umezawa ES, Luquetti AO, Levitus G, Ponce C, Ponce E, et al (2004) Serodiagnosis of chronic and acute Chagas’ disease with Trypanosoma cruzi recombinant proteins: results of a collaborative study in six Latin American countries. J Clin Microbiol 42: 449–452. Available: http://www.ncbi.nlm.nih.gov/pubmed/14715803. Accessed 1 August 2017. 10.1128/JCM.42.1.449-452.2004 14715803 PMC321695

[17] Granjon E, Dichtel-Danjoy M-L, Saba E, Sabino E, Campos de Oliveira L, et al (2016) Development of a Novel Multiplex Immunoassay Multi-cruzi for the Serological Confirmation of Chagas Disease. PLoS Negl Trop Dis 10: e0004596 Available: http://www.ncbi.nlm.nih.gov/pubmed/27035146. Accessed 28 July 2016. 10.1371/journal.pntd.0004596 27035146 PMC4818036

[18] Caballero ZC, Sousa OE, Marques WP, Saez-Alquezar A, Umezawa ES (2007) Evaluation of serological tests to identify Trypanosoma cruzi infection in humans and determine cross-reactivity with Trypanosoma rangeli and Leishmania spp. Clin Vaccine Immunol 14: 1045–1049. Available: http://www.pubmedcentral.nih.gov/articlerender.fcgi?artid=2044488&tool=pmcentrez&rendertype=abstract. Accessed 18 March 2015. 10.1128/CVI.00127-07 17522327 PMC2044488

[19] Reithinger R, Grijalva MJ, Chiriboga RF, de Noya BA, Torres JR, et al (2010) Rapid detection of Trypanosoma cruzi in human serum by use of an immunochromatographic dipstick test. J Clin Microbiol 48: 3003–3007. Available: http://www.pubmedcentral.nih.gov/articlerender.fcgi?artid=2916568&tool=pmcentrez&rendertype=abstract. Accessed 26 May 2015. 10.1128/JCM.02474-09 20534801 PMC2916568

[20] Cooley G, Etheridge RD, Boehlke C, Bundy B, Weatherly DB, et al (2008) High throughput selection of effective serodiagnostics for Trypanosoma cruzi infection. PLoS Negl Trop Dis 2: e316 Available: 10.1371/journal.pntd.0000316. Accessed 17 April 2015. 10.1371/journal.pntd.0000316 18841200 PMC2556098

[21] Noya O, Patarroyo ME, Guzmán F, Alarcón de Noya B (2003) Immunodiagnosis of parasitic diseases with synthetic peptides. Curr Protein Pept Sci 4: 299–308. Available: http://www.ncbi.nlm.nih.gov/pubmed/14529537. Accessed 26 May 2015. 14529537 10.2174/1389203033487153

[22] Yu Z, Carter JM, Huang SY, Lackland H, Sigal LH, et al Presentation of peptide antigens as albumin conjugates for use in detection of serum antibodies by enzyme-linked immunosorbent assay. Bioconjug Chem 7: 338–342. Available: http://www.ncbi.nlm.nih.gov/pubmed/8816957. Accessed 26 May 2015. 10.1021/bc960018s 8816957

[23] Houghton RL, Benson DR, Reynolds L, McNeill P, Sleath P, et al (2000) Multiepitope synthetic peptide and recombinant protein for the detection of antibodies to Trypanosoma cruzi in patients with treated or untreated Chagas’ disease. J Infect Dis 181: 325–330. Available: http://www.ncbi.nlm.nih.gov/pubmed/10608782. Accessed 26 May 2015. 10.1086/315165 10608782

[24] Houghton RL, Benson DR, Reynolds LD, McNeill PD, Sleath PR, et al (1999) A multi-epitope synthetic peptide and recombinant protein for the detection of antibodies to Trypanosoma cruzi in radioimmunoprecipitation-confirmed and consensus-positive sera. J Infect Dis 179: 1226–1234. Available: http://www.ncbi.nlm.nih.gov/pubmed/10191227. Accessed 26 May 2015. 10.1086/314723 10191227

[25] Ferreira AW, Belem ZR, Lemos EA, Reed SG, Campos-Neto A (2001) Enzyme-linked immunosorbent assay for serological diagnosis of Chagas’ disease employing a Trypanosoma cruzi recombinant antigen that consists of four different peptides. J Clin Microbiol 39: 4390–4395. Available: http://www.pubmedcentral.nih.gov/articlerender.fcgi?artid=88554&tool=pmcentrez&rendertype=abstract. Accessed 26 May 2015. 10.1128/JCM.39.12.4390-4395.2001 11724850 PMC88554

[26] Umezawa ES, Bastos SF, Coura JR, Levin MJ, Gonzalez A, et al (2003) An improved serodiagnostic test for Chagas’ disease employing a mixture of Trypanosoma cruzi recombinant antigens. Transfusion 43: 91–97. Available: http://www.ncbi.nlm.nih.gov/pubmed/12519436. Accessed 11 May 2015. 12519436 10.1046/j.1537-2995.2003.00279.x

[27] Camussone C, Gonzalez V, Belluzo MS, Pujato N, Ribone ME, et al (2009) Comparison of recombinant Trypanosoma cruzi peptide mixtures versus multiepitope chimeric proteins as sensitizing antigens for immunodiagnosis. Clin Vaccine Immunol 16: 899–905. Available: http://www.pubmedcentral.nih.gov/articlerender.fcgi?artid=2691048&tool=pmcentrez&rendertype=abstract. Accessed 26 May 2015. 10.1128/CVI.00005-09 19339486 PMC2691048

[28] Dai J, Jiang M, Wang Y, Qu L, Gong R, et al (2012) Evaluation of a recombinant multiepitope peptide for serodiagnosis of Toxoplasma gondii infection. Clin Vaccine Immunol 19: 338–342. Available: http://www.pubmedcentral.nih.gov/articlerender.fcgi?artid=3294622&tool=pmcentrez&rendertype=abstract. Accessed 26 May 2015. 10.1128/CVI.05553-11 22219311 PMC3294622

[29] Faria AR, de Castro Veloso L, Coura-Vital W, Reis AB, Damasceno LM, et al (2015) Novel recombinant multiepitope proteins for the diagnosis of asymptomatic leishmania infantum-infected dogs. PLoS Negl Trop Dis 9: e3429 Available: http://www.pubmedcentral.nih.gov/articlerender.fcgi?artid=4287523&tool=pmcentrez&rendertype=abstract. Accessed 26 May 2015. 10.1371/journal.pntd.0003429 25569685 PMC4287523

[30] Fournel S, Muller S (2003) Synthetic peptides in the diagnosis of systemic autoimmune diseases. Curr Protein Pept Sci 4: 261–274. Available: http://www.ncbi.nlm.nih.gov/pubmed/14529533. Accessed 26 May 2015. 14529533 10.2174/1389203033487126

[31] List C, Qi W, Maag E, Gottstein B, Müller N, et al (2010) Serodiagnosis of Echinococcus spp. infection: explorative selection of diagnostic antigens by peptide microarray. PLoS Negl Trop Dis 4: e771 Available: 10.1371/journal.pntd.0000771. 10.1371/journal.pntd.0000771 20689813 PMC2914747

[32] Coleman AS, Rossmann E, Yang X, Song H, Lamichhane CM, et al (2011) BBK07 immunodominant peptides as serodiagnostic markers of Lyme disease. Clin Vaccine Immunol 18: 406–413. Available: http://www.pubmedcentral.nih.gov/articlerender.fcgi?artid=3067378&tool=pmcentrez&rendertype=abstract. Accessed 26 May 2015. 10.1128/CVI.00461-10 21177911 PMC3067378

[33] Costa MM, Penido M, dos Santos MS, Doro D, de Freitas E, et al (2012) Improved canine and human visceral leishmaniasis immunodiagnosis using combinations of synthetic peptides in enzyme-linked immunosorbent assay. PLoS Negl Trop Dis 6: e1622 Available: http://www.pubmedcentral.nih.gov/articlerender.fcgi?artid=3358334&tool=pmcentrez&rendertype=abstract. Accessed 26 May 2015. 10.1371/journal.pntd.0001622 22629475 PMC3358334

[34] Menezes-Souza D, Mendes TA de O, Gomes M de S, Bartholomeu DC, Fujiwara RT (2015) Improving serodiagnosis of human and canine leishmaniasis with recombinant Leishmania braziliensis cathepsin l-like protein and a synthetic peptide containing its linear B-cell epitope. PLoS Negl Trop Dis 9: e3426 Available: http://www.pubmedcentral.nih.gov/articlerender.fcgi?artid=4287388&tool=pmcentrez&rendertype=abstract. Accessed 26 May 2015. 10.1371/journal.pntd.0003426 25569432 PMC4287388

[35] Carmona SJ, Sartor PA, Leguizamón MS, Campetella O, Agüero F (2012) Diagnostic peptide discovery: prioritization of pathogen diagnostic markers using multiple features. PLoS One 7: e50748 Available: 10.1371/journal.pone.0050748. 10.1371/journal.pone.0050748 23272069 PMC3522711

[36] Carmona SJ, Nielsen M, Schafer-Nielsen C, Mucci J, Altcheh J, et al (2015) Towards high-throughput immunomics for infectious diseases: use of next-generation peptide microarrays for rapid discovery and mapping of antigenic determinants. Mol Cell Proteomics 14: 1871–1884. Available: http://www.mcponline.org/cgi/content/long/M114.045906v1. Accessed 29 April 2015. 10.1074/mcp.M114.045906 25922409 PMC4587317

[37] Balouz V, Cámara M de LM, Cánepa GE, Carmona SJ, Volcovich R, et al (2015) Mapping Antigenic Motifs in the Trypomastigote Small Surface Antigen from Trypanosoma cruzi. Clin Vaccine Immunol 22: 304–312. Available: http://www.ncbi.nlm.nih.gov/pubmed/25589551. Accessed 16 March 2015. 10.1128/CVI.00684-14 25589551 PMC4340888

[38] Bartholomeu DC, Cerqueira GC, Leão ACA, DaRocha WD, Pais FS, et al (2009) Genomic organization and expression profile of the mucin-associated surface protein (masp) family of the human pathogen Trypanosoma cruzi. Nucleic Acids Res 37: 3407–3417. Available: 10.1093/nar/gkp172. 10.1093/nar/gkp172 19336417 PMC2691823

[39] Ibañez CF, Affranchino JL, Frasch AC (1987) Antigenic determinants of Trypanosoma cruzi defined by cloning of parasite DNA. Mol Biochem Parasitol 25: 175–184. Available: http://www.ncbi.nlm.nih.gov/pubmed/2444885. Accessed 17 April 2015. 2444885 10.1016/0166-6851(87)90006-5

[40] Santos FLN, Celedon PAF, Zanchin NIT, Brasil T de AC, Foti L, et al (2016) Performance Assessment of Four Chimeric Trypanosoma cruzi Antigens Based on Antigen-Antibody Detection for Diagnosis of Chronic Chagas Disease. PLoS One 11: e0161100 Available: http://dx.plos.org/10.1371/journal.pone.0161100. Accessed 16 August 2016. 10.1371/journal.pone.0161100 27517281 PMC4982698

[41] Buschiazzo A, Campetella OE, Macina RA, Salceda S, Frasch ACC, et al (1992) Sequence of the gene for a Trypanosoma cruzi protein antigenic during the chronic phase of human Chagas disease. Mol Biochem Parasitol 54: 125–128. Available: http://www.sciencedirect.com/science/article/pii/016668519290105S. Accessed 17 April 2015. 1518528 10.1016/0166-6851(92)90105-s

[42] Campetella O, Sánchez D, Cazzulo JJ, Frasch AC (1992) A superfamily of Trypanosoma cruzi surface antigens. Parasitol Today 8: 378–381. Available: http://www.ncbi.nlm.nih.gov/pubmed/15463546. Accessed 17 April 2015. 15463546 10.1016/0169-4758(92)90175-2

[43] Alvarez P, Leguizamón MS, Buscaglia CA, Pitcovsky TA, Campetella O, et al (2001) Multiple overlapping epitopes in the repetitive unit of the shed acute-phase antigen from Trypanosoma cruzi enhance its immunogenic properties. Infect Immun 69: 7946–7949. Available: http://www.ncbi.nlm.nih.gov/pubmed/11705983. Accessed 13 January 2017. 10.1128/IAI.69.12.7946-7949.2001 11705983 PMC98897

[44] Buscaglia CA, Campo VA, Di Noia JM, Torrecilhas ACT, De Marchi CR, et al (2004) The surface coat of the mammal-dwelling infective trypomastigote stage of Trypanosoma cruzi is formed by highly diverse immunogenic mucins. J Biol Chem 279: 15860–15869. Available: http://www.ncbi.nlm.nih.gov/pubmed/14749325. Accessed 29 December 2014. 10.1074/jbc.M314051200 14749325

[45] Ibañez CF, Affranchino JL, Macina RA, Reyes MB, Leguizamon S, et al (1988) Multiple Trypanosoma cruzi antigens containing tandemly repeated amino acid sequence motifs. Mol Biochem Parasitol 30: 27–33. Available: http://www.ncbi.nlm.nih.gov/pubmed/3135494. Accessed 6 January 2017. 3135494 10.1016/0166-6851(88)90129-6

[46] Goto Y, Carter D, Reed SG (2008) Immunological dominance of Trypanosoma cruzi tandem repeat proteins. Infect Immun 76: 3967–3974. Available: http://iai.asm.org/content/76/9/3967.full. Accessed 17 April 2015. 10.1128/IAI.00604-08 18625739 PMC2519453

[47] Pérez CL, Larsen M V., Gustafsson R, Norström MM, Atlas A, et al (2008) Broadly immunogenic HLA class I supertype-restricted elite CTL epitopes recognized in a diverse population infected with different HIV-1 subtypes. J Immunol 180: 5092–5100. Available: http://www.jimmunol.org/content/180/7/5092. Accessed 26 May 2015. 18354235 10.4049/jimmunol.180.7.5092

[48] Orlowski M, Meister A (1971) 6 Enzymology of Pyrrolidone Carboxylic Acid. Enzym 4: 123–151. 10.1016/S1874-6047(08)60366-2

[49] Campo VA, Buscaglia CA, Di Noia JM, Frasch ACC (2006) Immunocharacterization of the mucin-type proteins from the intracellular stage of Trypanosoma cruzi. Microbes Infect 8: 401–409. Available: http://www.ncbi.nlm.nih.gov/pubmed/16253534. Accessed 6 January 2017. 10.1016/j.micinf.2005.07.008 16253534

[50] Marco JD, Barroso PA, Mimori T, Locatelli FM, Tomatani A, et al (2012) Polymorphism-specific PCR enhances the diagnostic performance of American tegumentary leishmaniasis and allows the rapid identification of Leishmania species from Argentina. BMC Infect Dis 12: 191 Available: http://www.ncbi.nlm.nih.gov/pubmed/22894734. Accessed 3 July 2017. 10.1186/1471-2334-12-191 22894734 PMC3449195

[51] Agresti A, Franklin CA (2013) Statistics: the art and science of learning from data Pearson. pp. 348–399.

[52] Greiner M, Pfeiffer D, Smith RD (2000) Principles and practical application of the receiver-operating characteristic analysis for diagnostic tests. Prev Vet Med 45: 23–41. Available: http://www.ncbi.nlm.nih.gov/pubmed/10802332. Accessed 13 January 2017. 10802332 10.1016/s0167-5877(00)00115-x

[53] Sing T, Sander O, Beerenwinkel N, Lengauer T (2005) ROCR: visualizing classifier performance in R. Bioinformatics 21: 3940–3941. Available: 10.1093/bioinformatics/bti623. 10.1093/bioinformatics/bti623 16096348

[54] Frasch AC, Cazzulo JJ, Aslund L, Pettersson U (1991) Comparison of genes encoding Trypanosoma cruzi antigens. Parasitol Today 7: 148–151. 15463477 10.1016/0169-4758(91)90284-u

[55] Leguizamón MS, Russomando G, de Arias AR, Samudio M, Cabral M, et al (1998) Use of trans-sialidase inhibition assay in a population serologically negative for Trypanosoma cruzi but at a high risk of infection. Clin Diagn Lab Immunol 5: 254–255. 9521153 10.1128/cdli.5.2.254-255.1998 PMC121368

[56] Bottino CG, Gomes LP, Pereira JB, Coura JR, Provance DW, et al (2013) Chagas disease-specific antigens: characterization of epitopes in CRA/FRA by synthetic peptide mapping and evaluation by ELISA-peptide assay. BMC Infect Dis 13: 568 Available: http://www.pubmedcentral.nih.gov/articlerender.fcgi?artid=3890492&tool=pmcentrez&rendertype=abstract. Accessed 26 May 2015. 10.1186/1471-2334-13-568 24299278 PMC3890492

[57] Pitcovsky TA, Mucci J, Alvarez P, Leguizamon MS, Burrone O, et al (2001) Epitope Mapping of trans-Sialidase from Trypanosoma cruzi Reveals the Presence of Several Cross-Reactive Determinants. Infect Immun 69: 1869–1875. Available: http://www.ncbi.nlm.nih.gov/pubmed/11179365. Accessed 1 August 2017. 10.1128/IAI.69.3.1869-1875.2001 11179365 PMC98094

[58] dos Santos SL, Freitas LM, Lobo FP, Rodrigues-Luiz GF, de Oliveira Mendes TA, et al (2012) The MASP family of Trypanosoma cruzi: changes in gene expression and antigenic profile during the acute phase of experimental infection. PLoS Negl Trop Dis 6: e1779 Available: http://www.ncbi.nlm.nih.gov/pubmed/22905275. Accessed 1 August 2017. 10.1371/journal.pntd.0001779 22905275 PMC3419193

[59] Durante I, La Spina P, Carmona SJ, Agüero F, Buscaglia CA (2017) High-resolution profiling of linear B-cell epitopes from mucin-associated surface proteins (MASPs) of Trypanosoma cruzi during human infections. PLoS Negl Trop Dis 11: e0005986 10.1371/journal.pntd.0005986 28961244 PMC5636173

[60] Campo V, Di Noia JM, Buscaglia CA, Agüero F, Sánchez DO, et al (2004) Differential accumulation of mutations localized in particular domains of the mucin genes expressed in the vertebrate host stage of Trypanosoma cruzi. Mol Biochem Parasitol 133: 81–91. 10.1016/j.molbiopara.2003.09.006 14668015

[61] Burns JM, Shreffler WG, Rosman DE, Sleath PR, March CJ, et al (1992) Identification and synthesis of a major conserved antigenic epitope of Trypanosoma cruzi. Proc Natl Acad Sci U S A 89: 1239–1243. Available: http://www.pubmedcentral.nih.gov/articlerender.fcgi?artid=48424&tool=pmcentrez&rendertype=abstract. Accessed 17 April 2015. 1371355 10.1073/pnas.89.4.1239 PMC48424

[62] Affranchino JL, Ibañez CF, Luquetti AO, Rassi A, Reyes MB, et al (1989) Identification of a Trypanosoma cruzi antigen that is shed during the acute phase of Chagas’ disease. Mol Biochem Parasitol 34: 221–228. Available: http://www.ncbi.nlm.nih.gov/pubmed/2499788. Accessed 17 April 2015. 2499788 10.1016/0166-6851(89)90050-9

[63] Anti-Trypanosoma cruzi Assays: WHO Library Cataloguing-in-Publication Data (2010). Available: http://www.who.int/diagnostics_laboratory/publications/anti_t_cruzi_assays.pdf. Accessed 1 August 2017.

[64] El-Sayed NM, Myler PJ, Bartholomeu DC, Nilsson D, Aggarwal G, et al (2005) The genome sequence of Trypanosoma cruzi, etiologic agent of Chagas disease. Science (80-) 309: 409–415. Available: 10.1126/science.1112631. 16020725

[65] Franzén O, Ochaya S, Sherwood E, Lewis MD, Llewellyn MS, et al (2011) Shotgun sequencing analysis of Trypanosoma cruzi I Sylvio X10/1 and comparison with T. cruzi VI CL Brener. PLoS Negl Trop Dis 5: e984 Available: http://www.pubmedcentral.nih.gov/articlerender.fcgi?artid=3050914&tool=pmcentrez&rendertype=abstract. Accessed 9 December 2014. 10.1371/journal.pntd.0000984 21408126 PMC3050914

[66] Aslett M, Aurrecoechea C, Berriman M, Brestelli J, Brunk BP, et al (2010) TriTrypDB: a functional genomic resource for the Trypanosomatidae. Nucleic Acids Res 38: D457—D462. Available: 10.1093/nar/gkp851. 10.1093/nar/gkp851 19843604 PMC2808979

[67] Ferreira LR, Kesper N, Teixeira MMG, Laurenti MD, Barbieri CL, et al (2014) New insights about cross-reactive epitopes of six trypanosomatid genera revealed that Crithidia and Leptomonas have antigenic similarity to L. (L.) chagasi. Acta Trop 131: 41–46. Available: http://www.ncbi.nlm.nih.gov/pubmed/24275757. Accessed 1 August 2017. 10.1016/j.actatropica.2013.11.010 24275757

