# Supplementary material for: Next-generation ELISA diagnostic assay for Chagas Disease based on the combination of short peptidic epitopes
Source: PLoS Negl Trop Dis. 2017 Oct 9;11(10):e0005972. doi: 10.1371/journal.pntd.0005972 (PMC5648266; doi:10.1371/journal.pntd.0005972)
Supplement: S1 Fig — The antibody binding profiles of antigens were derived from previously published data [36](ArrayExpress accession number E-MTAB-3008). Briefly, high-density peptide microarray slides were assayed with purified immunoglobulins from healthy subjects (four pools of samples labeled A-D, shown as dashed lines) or Chagas positive subjects (four pools of samples labeled A-D, shown as solid lines). Antibody binding profiles were reconstructed for each of the selected antigens as described previously. Each plot in the figure shows the normalized and smoothed signal profile for a single antigen (one per page). A different scale may be used in each plot to best accommodate all peaks. The location of each the selected peptides used in this study is shown in context with other antigenic regions in each antigen. File: S1 Fig. (PDF) [file pntd.0005972.s001.pdf]

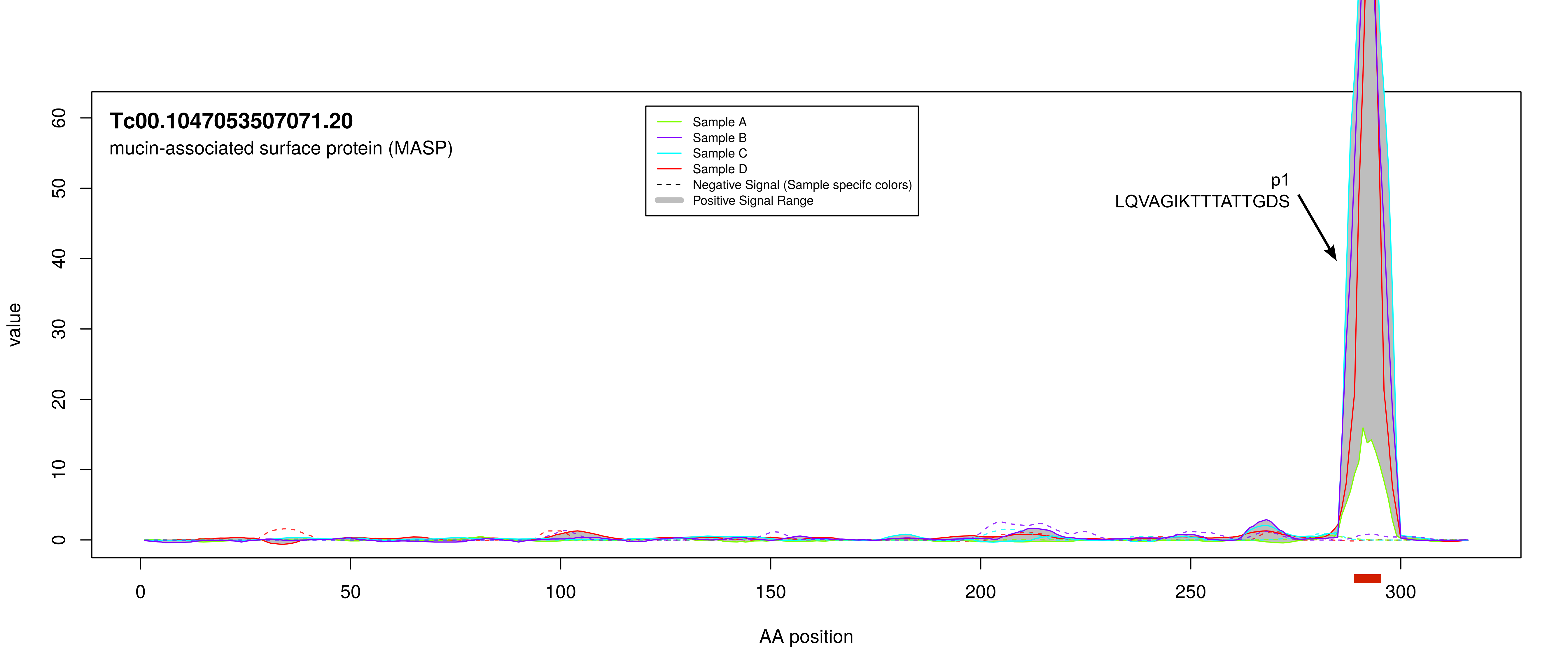
Tc00.1047053507071.20
mucin-associated surface protein (MASP)
Sample A
Sample B
Sample C
Sample D
Negative Signal (Sample specifc colors)
Positive Signal Range
p1
LQVAGIKTTTATTGDS
value
AA position
0
10
20
30
40
50
60
0
50
100
150
200
250
300

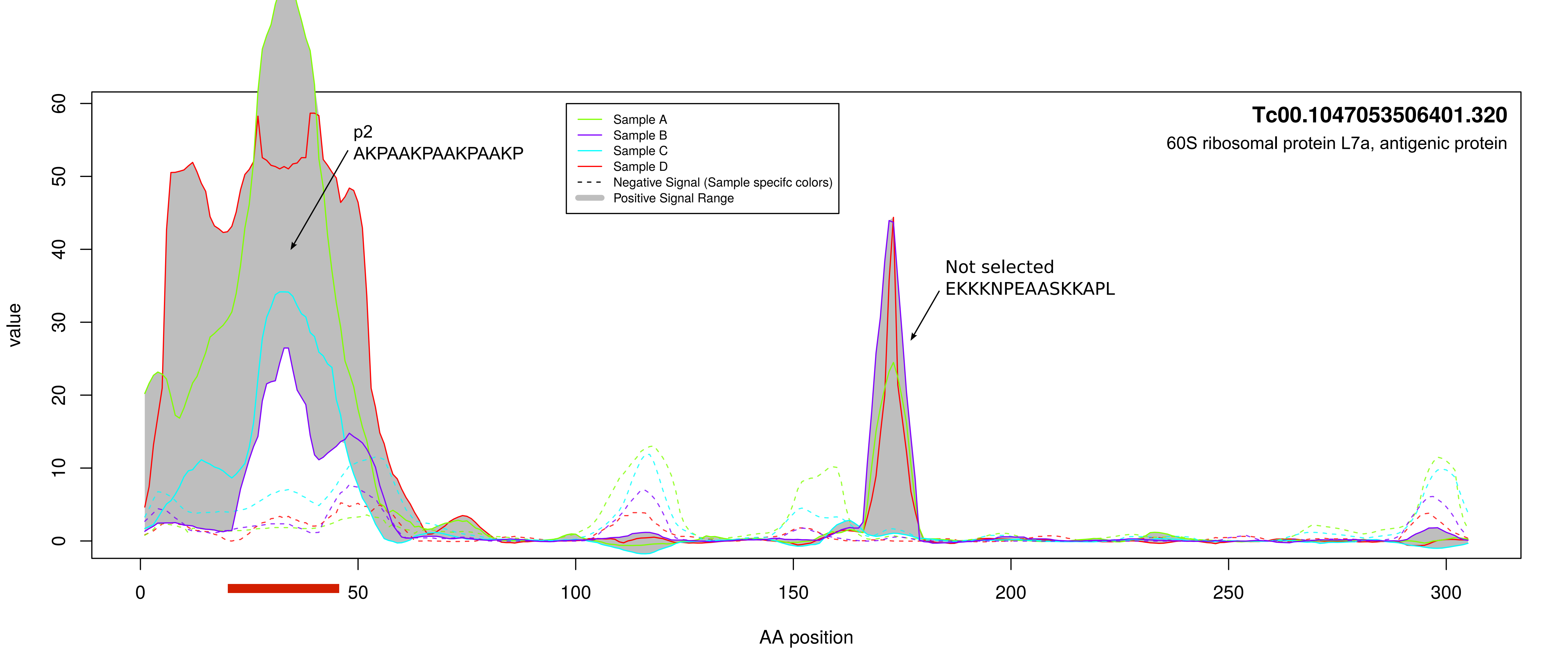
Tc00.1047053506401.320
60S ribosomal protein L7a, antigenic protein
Sample A
Sample B
Sample C
Sample D
Negative Signal (Sample specifc colors)
Positive Signal Range
p2
AKPAAKPAAKPAAKP
Not selected
EKKKNPEAASKKAPL
value
AA position
0
10
20
30
40
50
60
0
50
100
150
200
250
300

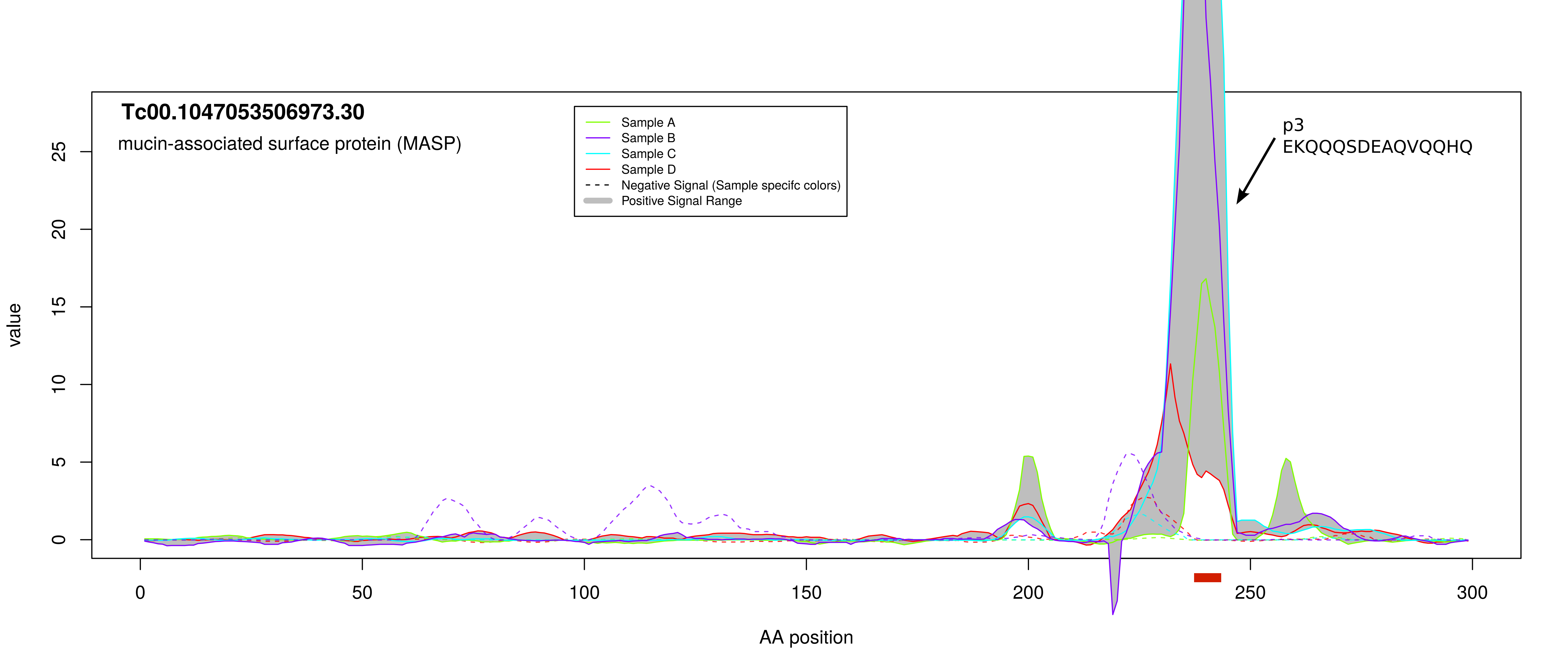

Tc00.1047053506973.30
mucin-associated surface protein (MASP)
Sample A
Sample B
Sample C
Sample D
Negative Signal (Sample specifc colors)
Positive Signal Range
p3
EKQQQSDEAQVQQHQ
value
0
5
10
15
20
25
AA position
0
50
100
150
200
250
300

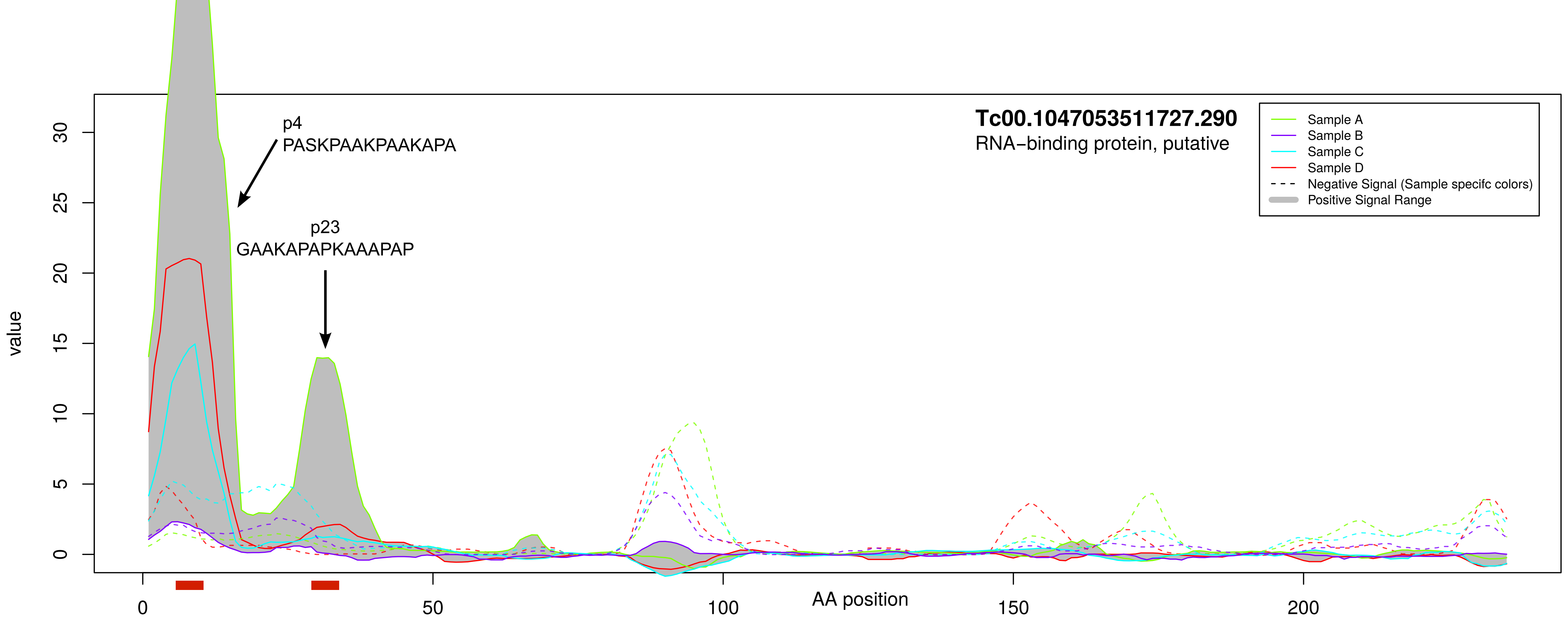

Tc00.1047053511727.290
RNA-binding protein, putative
Sample A
Sample B
Sample C
Sample D
Negative Signal (Sample specifc colors)
Positive Signal Range
p4
PASKPAAKPAAKAPA
p23
GAAKAPAPKAAAPAP
value
AA position
0
5
10
15
20
25
30
0
50
100
150
200

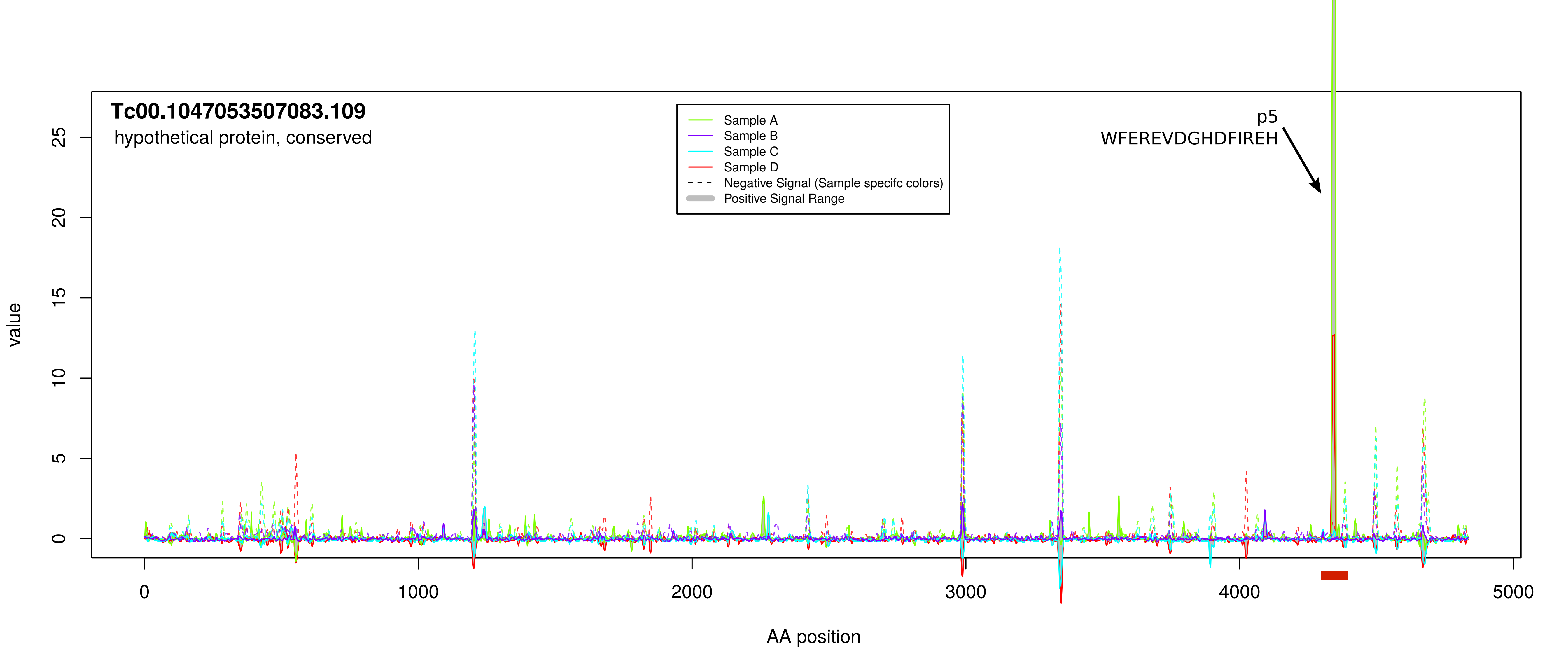
Tc00.1047053507083.109
hypothetical protein, conserved
Sample A
Sample B
Sample C
Sample D
Negative Signal (Sample specifc colors)
Positive Signal Range
p5
WFEREVDGHDFIREH
value
AA position
0
5
10
15
20
25
0
1000
2000
3000
4000
5000

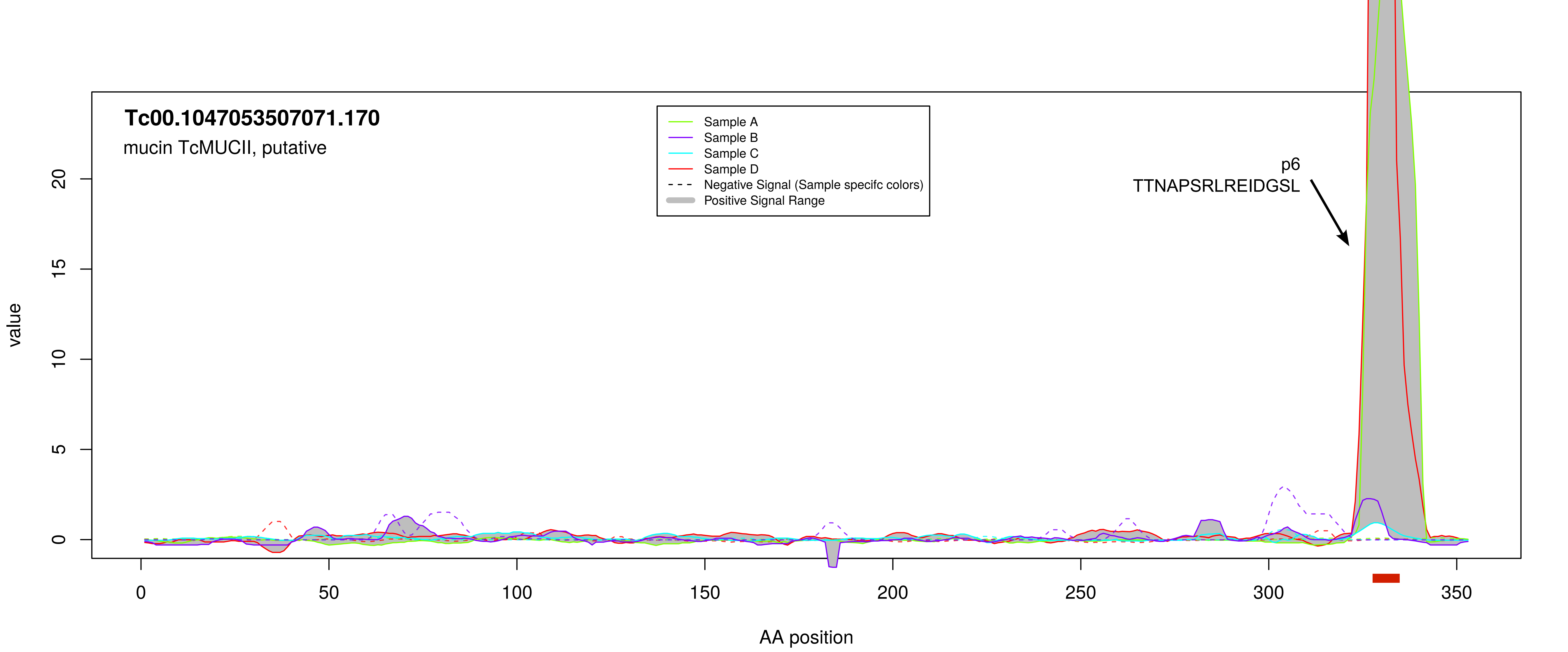

Tc00.1047053507071.170
mucin TcMUCII, putative
Sample A
Sample B
Sample C
Sample D
Negative Signal (Sample specifc colors)
Positive Signal Range
p6
TTNAPSRLREIDGSL
value
AA position
0
5
10
15
20
0
50
100
150
200
250
300
350

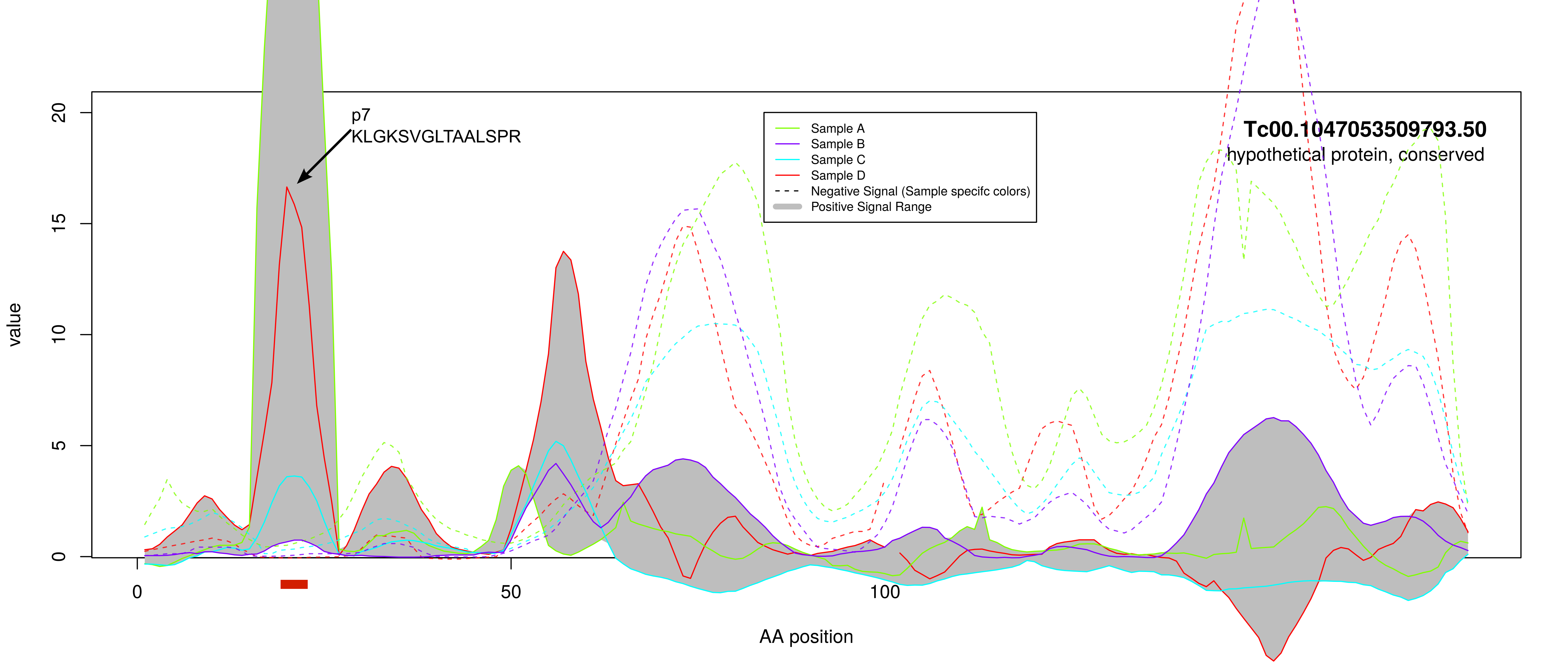
p7
KLGKSVGLTAALSPR
Sample A
Sample B
Sample C
Sample D
Negative Signal (Sample specifc colors)
Positive Signal Range
Tc00.1047053509793.50
hypothetical protein, conserved
value
AA position
0
5
10
15
20
0
50
100

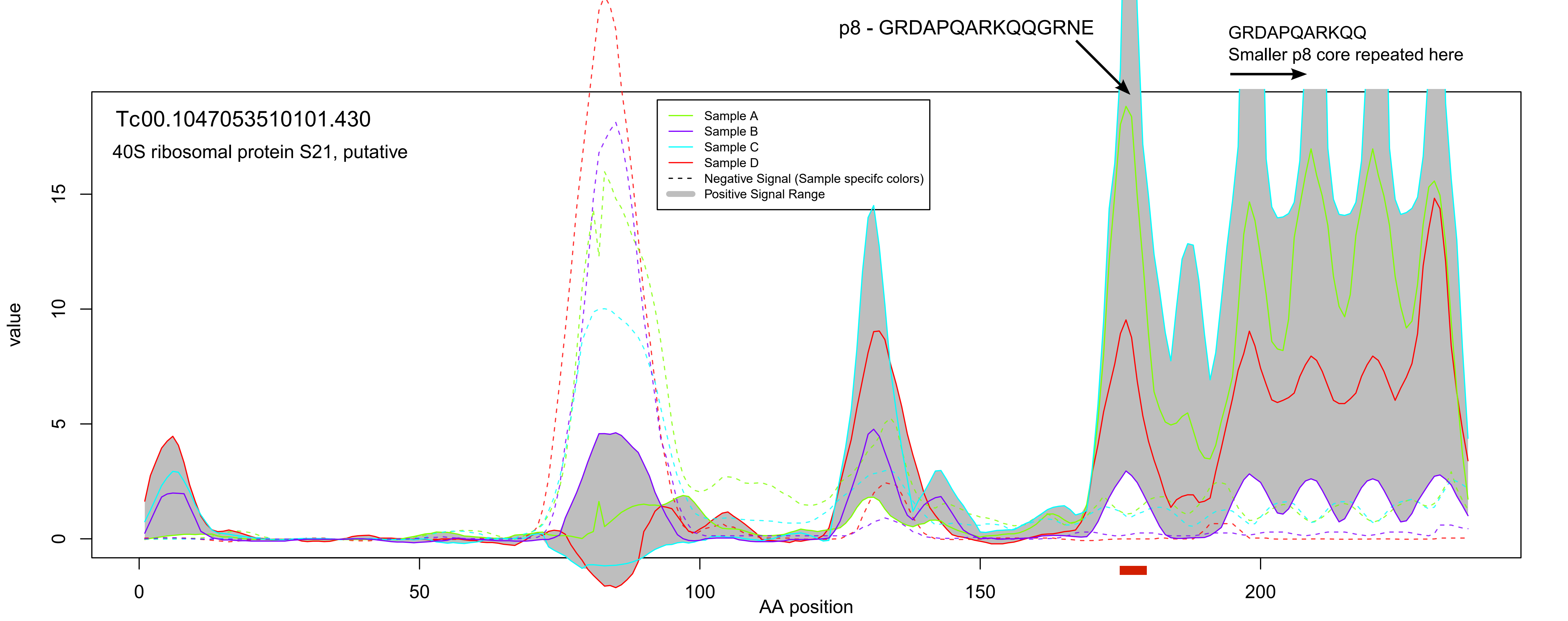

Tc00.1047053510101.430
40S ribosomal protein S21, putative
Sample A
Sample B
Sample C
Sample D
Negative Signal (Sample specifc colors)
Positive Signal Range
p8 - GRDAPQARKQQGRNE
GRDAPQARKQQ
Smaller p8 core repeated here
value
AA position
0
5
10
15
0
50
100
150
200

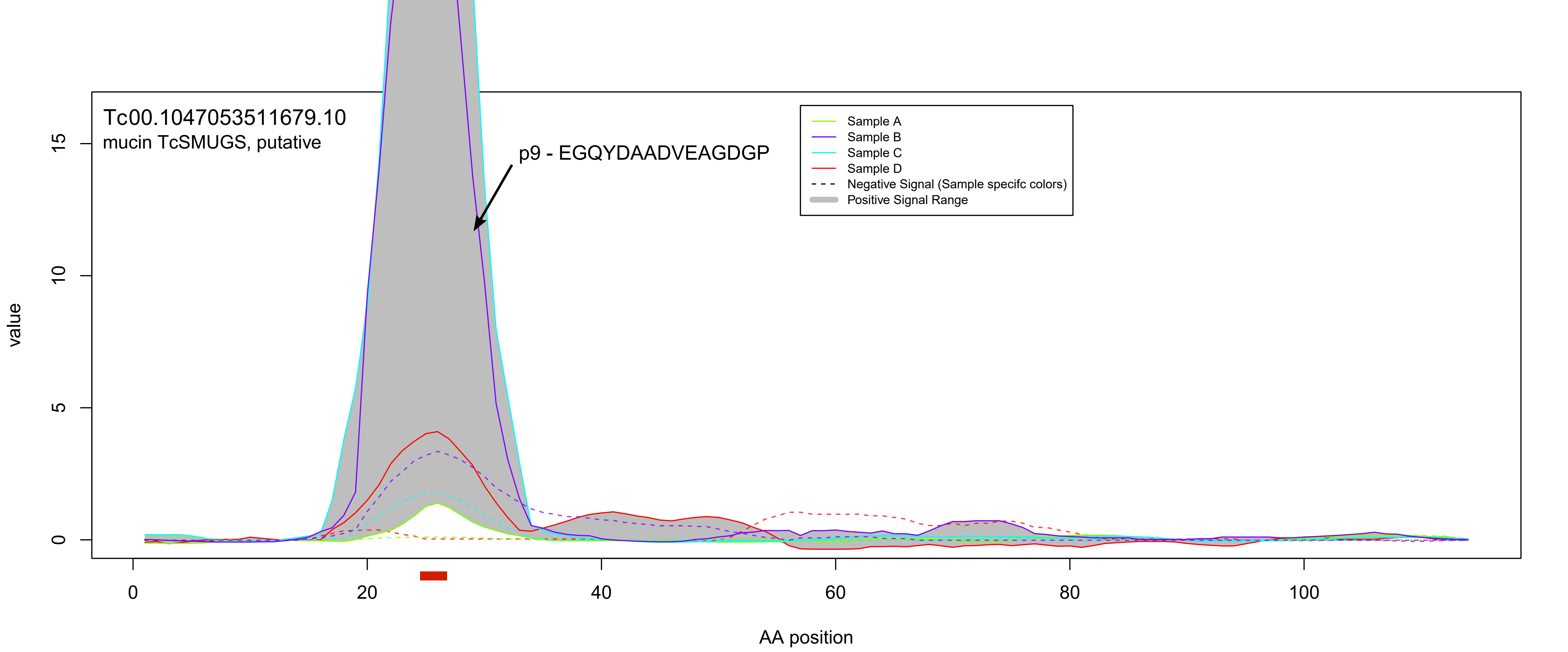

Tc00.1047053511679.10
mucin TcSMUGS, putative
p9 - EGQYDAADVEAGDGP
Sample A
Sample B
Sample C
Sample D
Negative Signal (Sample specifc colors)
Positive Signal Range
value
AA position
0
5
10
15
0
20
40
60
80
100

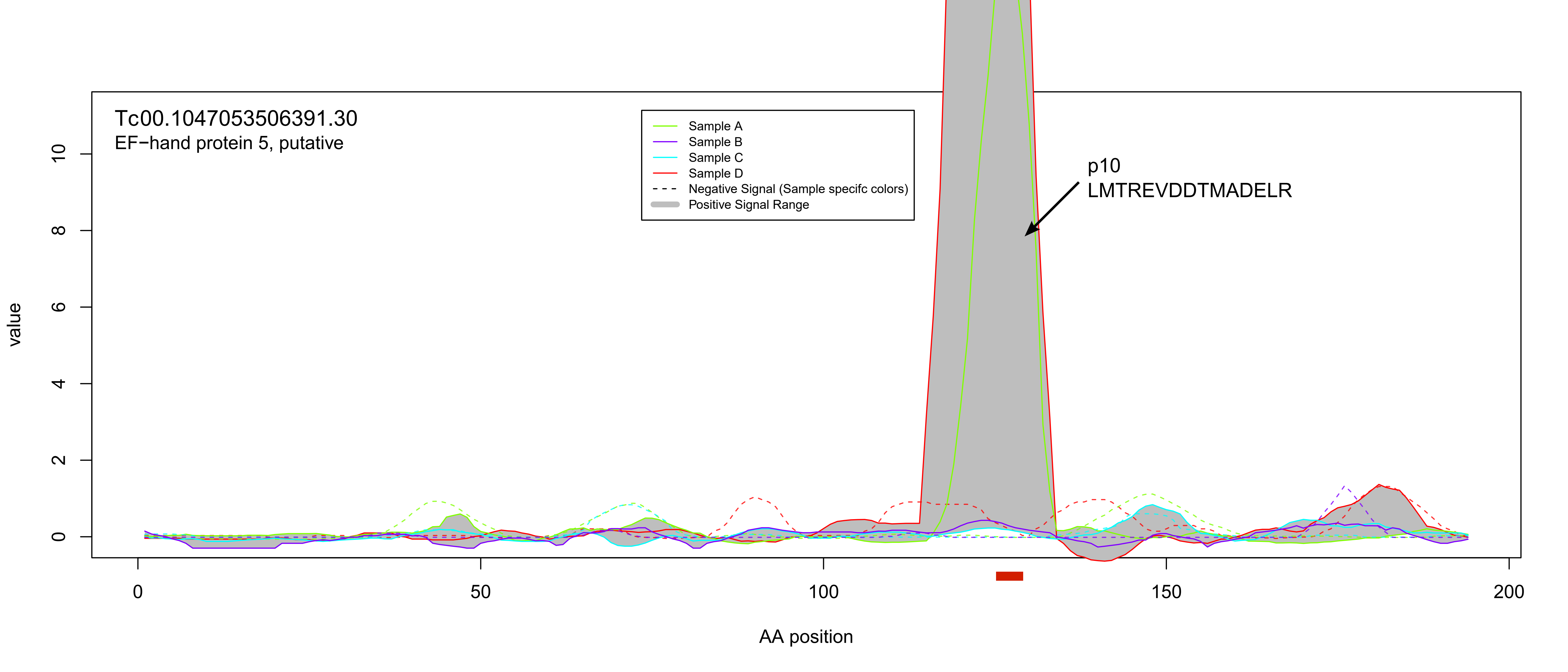

Tc00.1047053506391.30
EF−hand protein 5, putative
Sample A
Sample B
Sample C
Sample D
Negative Signal (Sample specifc colors)
Positive Signal Range
p10
LMTREVDDTMADELR
value
0
2
4
6
8
10
0
50
100
150
200
AA position

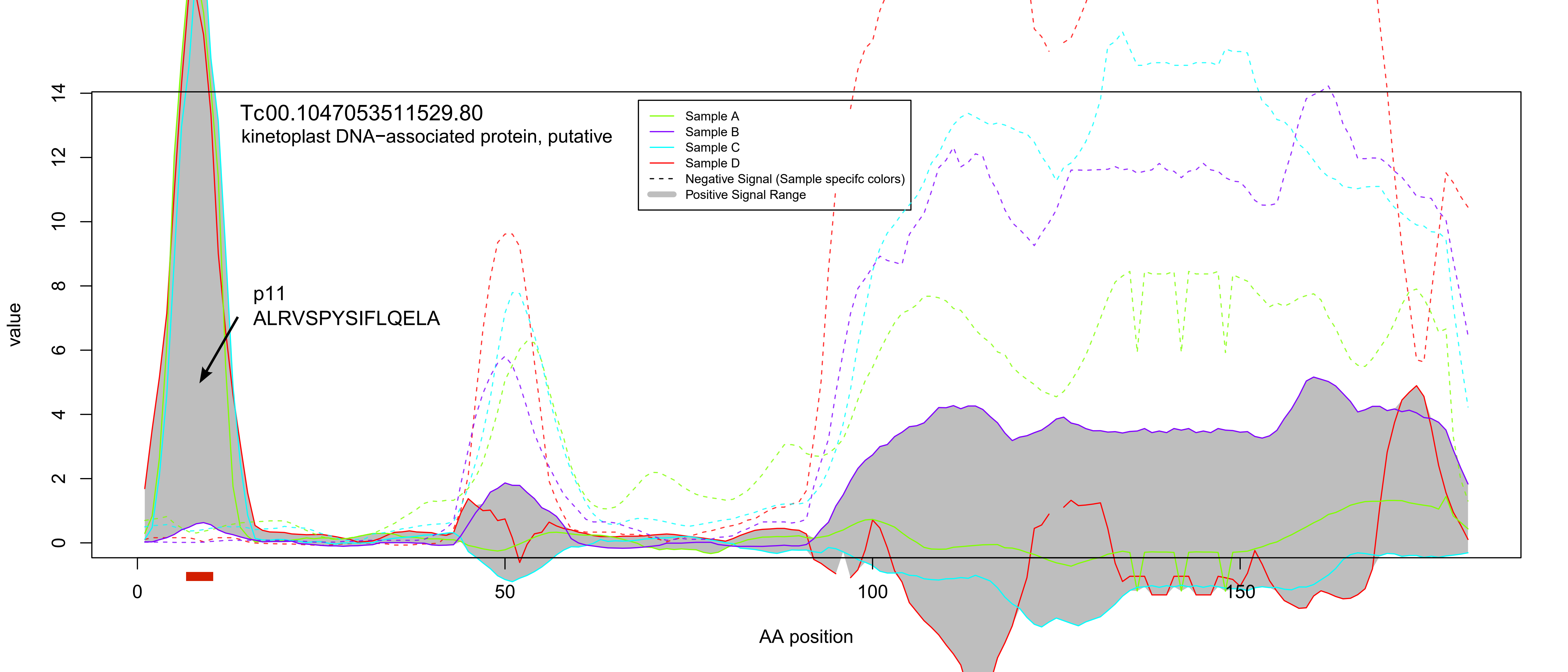

Tc00.1047053511529.80
kinetoplast DNA−associated protein, putative
p11
ALRVSPYSIFLQELA
Sample A
Sample B
Sample C
Sample D
Negative Signal (Sample specifc colors)
Positive Signal Range
value
AA position
0
2
4
6
8
10
12
14
0
50
100
150

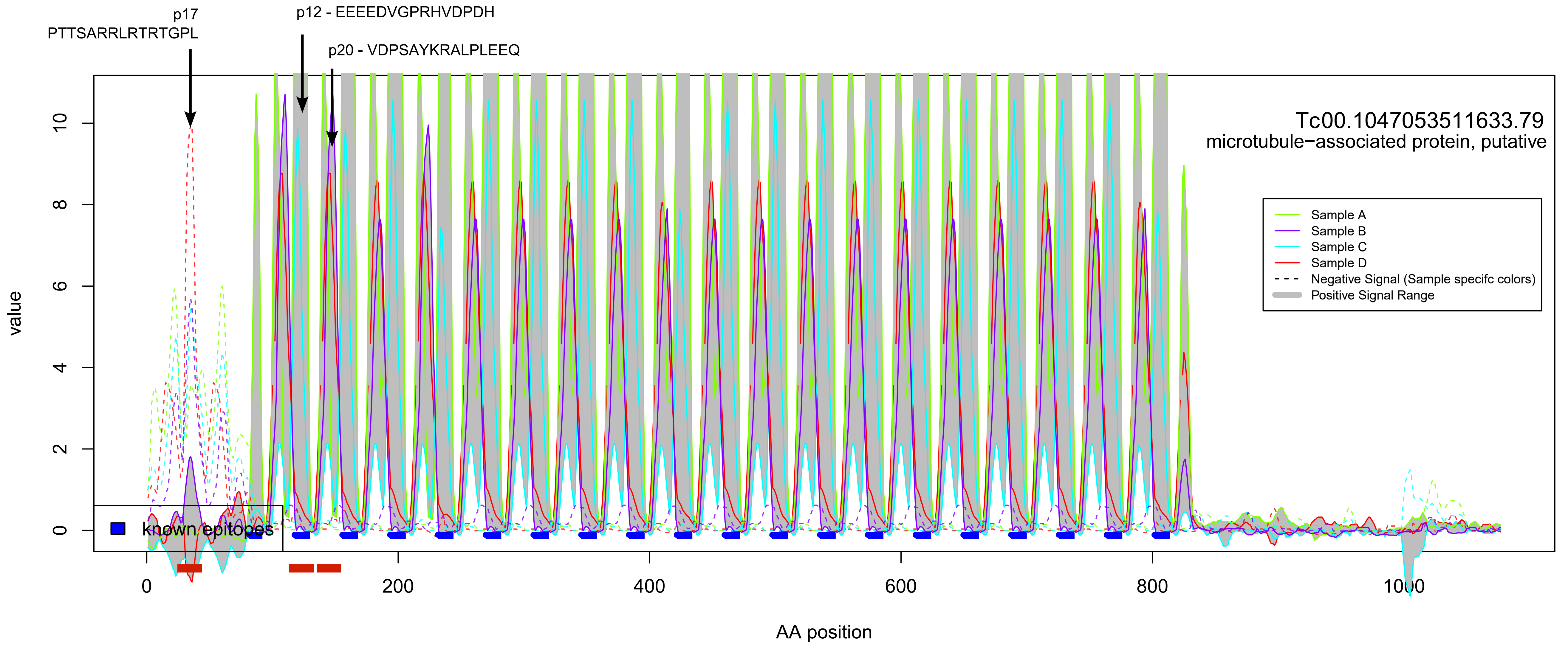

p17
PTTSARRLRTRTGPL
p12 - EEEEDVGPRHVDPDH
p20 - VDPSAYKRALPLEEQ
Tc00.1047053511633.79
microtubule−associated protein, putative
Sample A
Sample B
Sample C
Sample D
Negative Signal (Sample specifc colors)
Positive Signal Range
known epitopes
value
AA position
0
2
4
6
8
10
0
200
400
600
800
1000

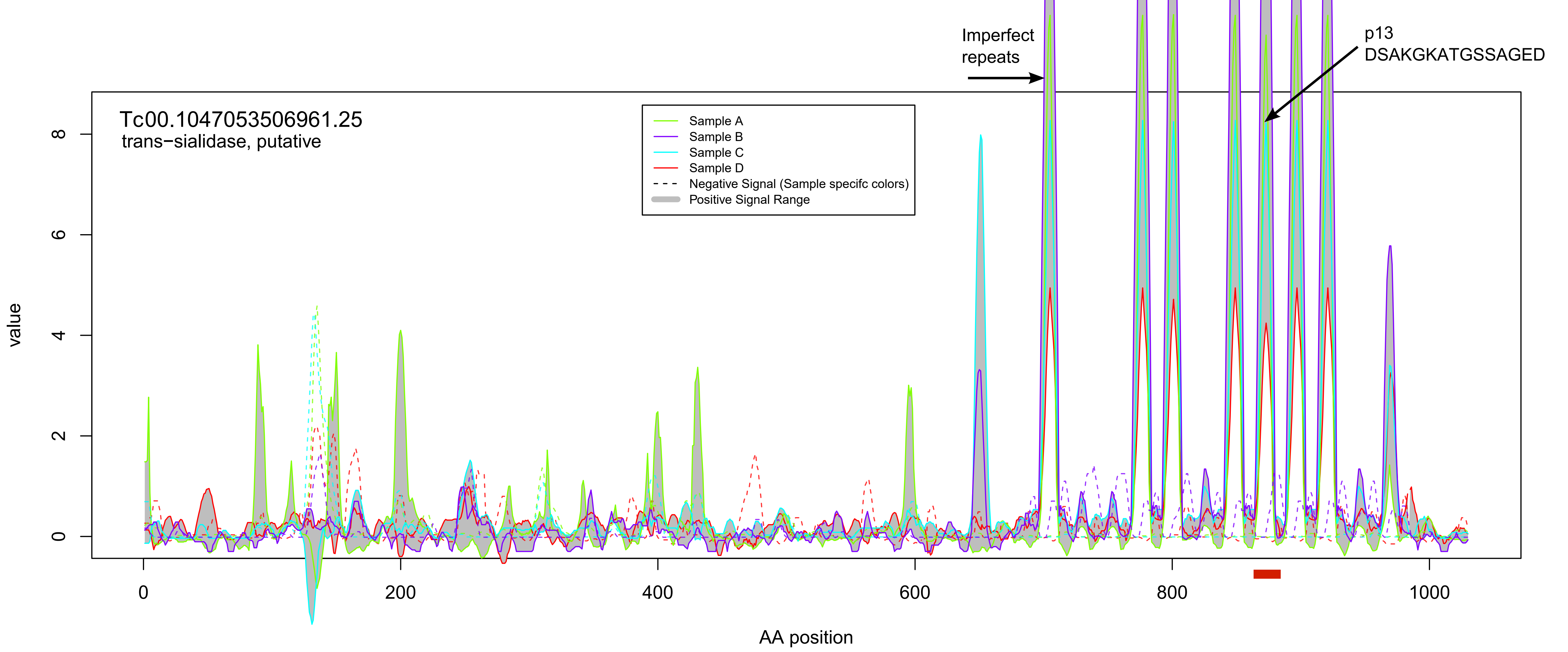

Imperfect repeats
p13
DSAKGKATGSSAGED
Tc00.1047053506961.25
trans−sialidase, putative
Sample A
Sample B
Sample C
Sample D
Negative Signal (Sample specifc colors)
Positive Signal Range
value
AA position
0
2
4
6
8
0
200
400
600
800
1000

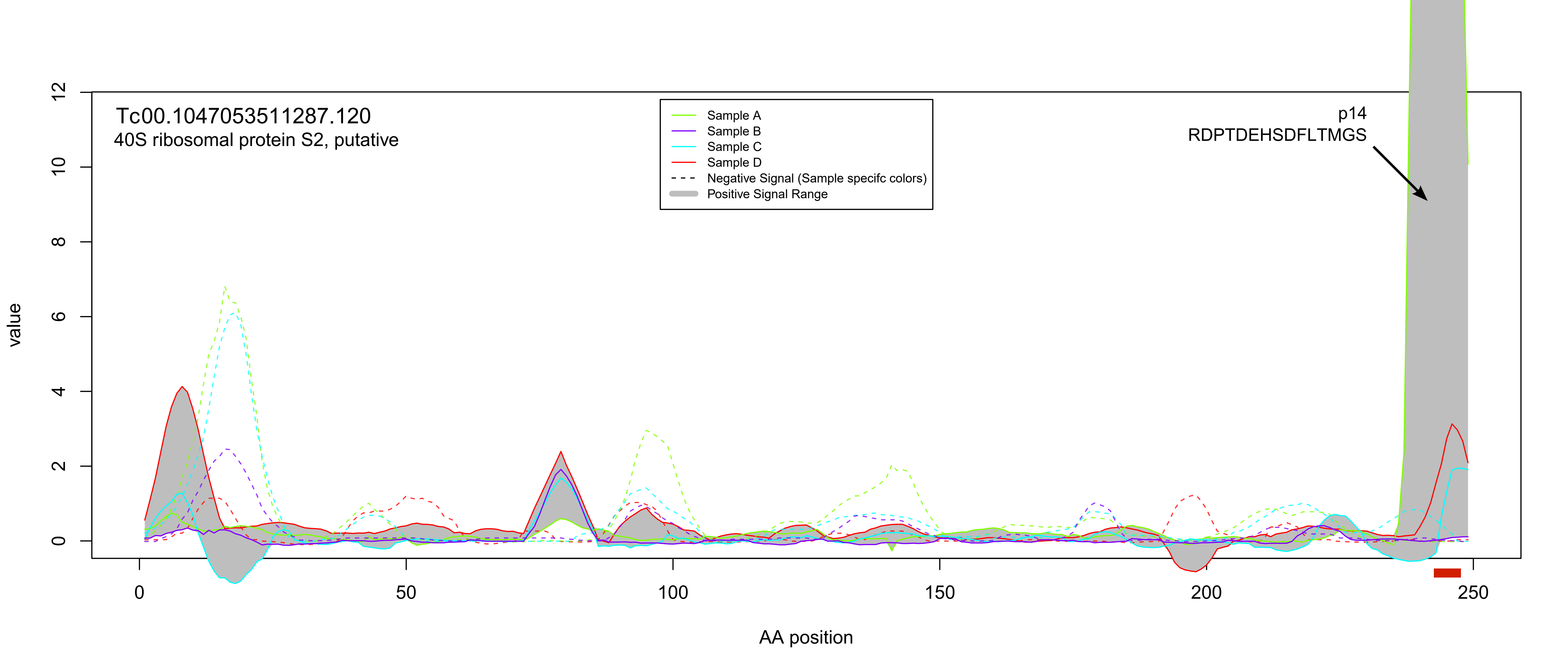
Tc00.1047053511287.120
40S ribosomal protein S2, putative
Sample A
Sample B
Sample C
Sample D
Negative Signal (Sample specifc colors)
Positive Signal Range
p14
RDPTDEHSDFLTMGS
value
AA position
0
2
4
6
8
10
12
0
50
100
150
200
250

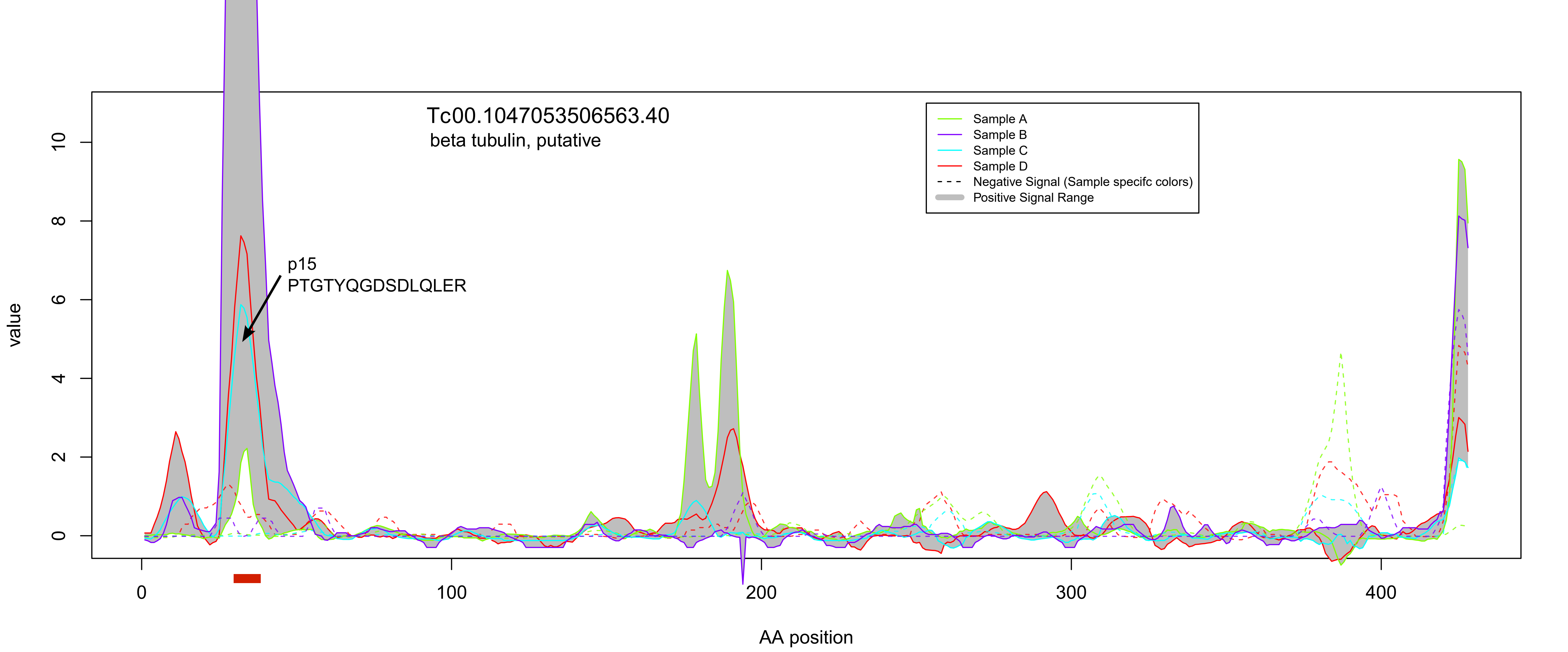

Tc00.1047053506563.40
beta tubulin, putative
Sample A
Sample B
Sample C
Sample D
Negative Signal (Sample specifc colors)
Positive Signal Range
p15
PTGTYQGDSDLQLER
value
0
2
4
6
8
10
0
100
200
300
400
AA position

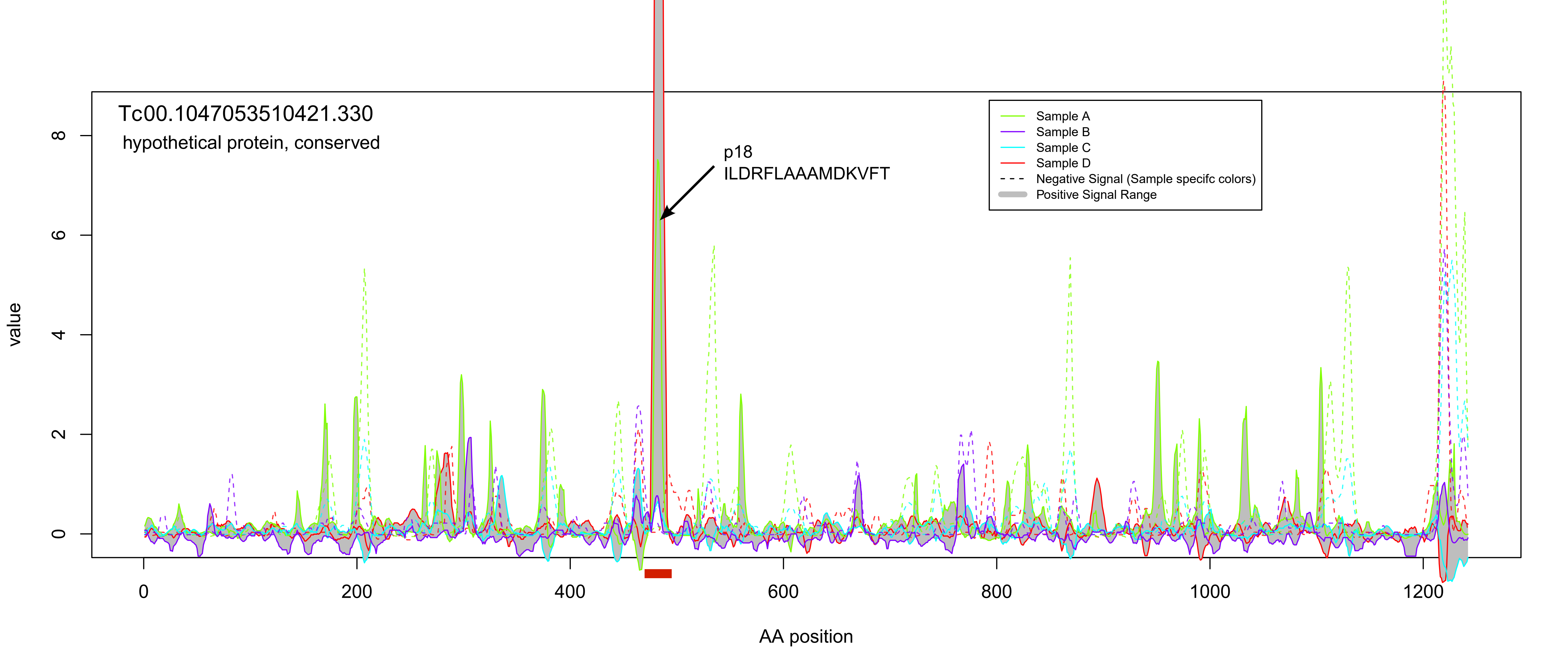

Tc00.1047053510421.330
hypothetical protein, conserved
p18
ILDRFLAAAMDKVFT
Sample A
Sample B
Sample C
Sample D
Negative Signal (Sample specifc colors)
Positive Signal Range
value
AA position
0
2
4
6
8
0
200
400
600
800
1000
1200

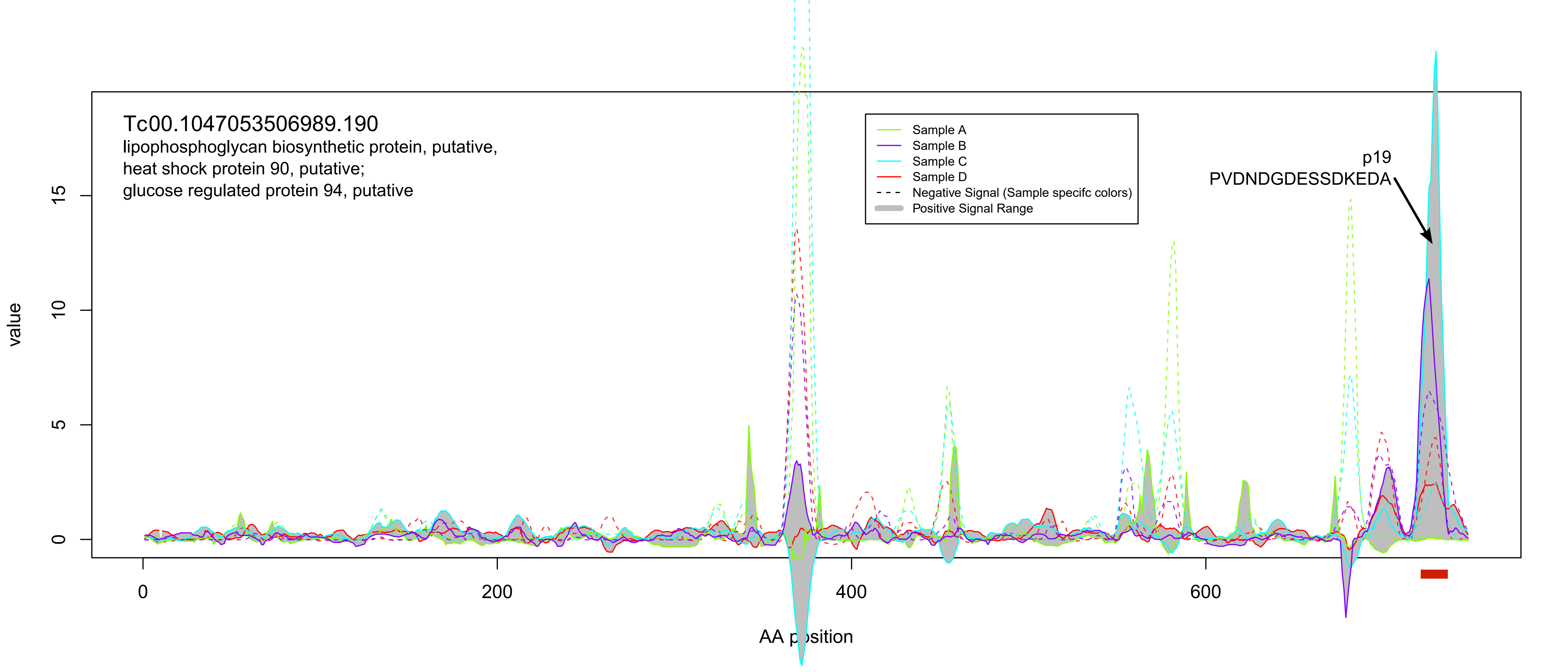

Tc00.1047053506989.190
lipophosphoglycan biosynthetic protein, putative,
heat shock protein 90, putative;
glucose regulated protein 94, putative
Sample A
Sample B
Sample C
Sample D
Negative Signal (Sample specifc colors)
Positive Signal Range
p19
PVDNDGDESSDKEDA
value
0
5
10
15
0
200
400
600
AA position

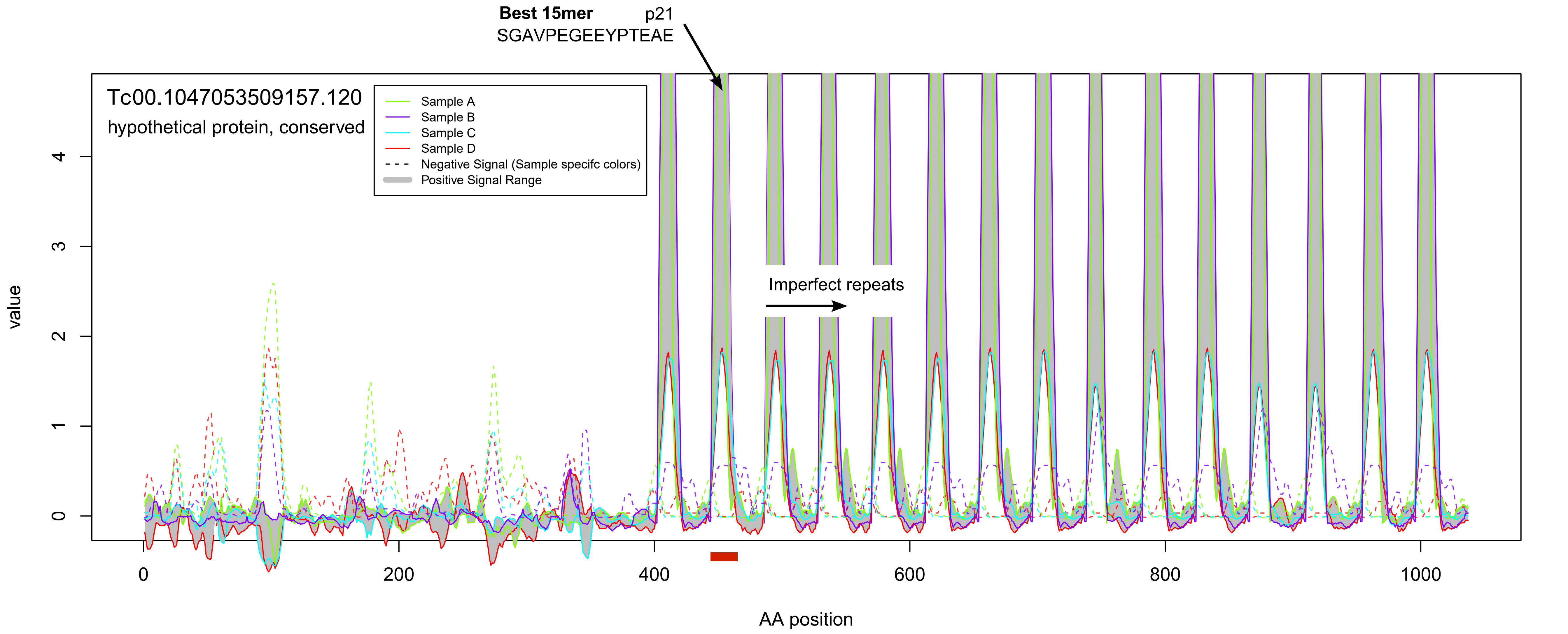
Best 15mer
p21
SGAVPEGEEYPTEAE
Tc00.1047053509157.120
hypothetical protein, conserved
Sample A
Sample B
Sample C
Sample D
Negative Signal (Sample specifc colors)
Positive Signal Range
Imperfect repeats
value
0
1
2
3
4
0
200
400
600
800
1000
AA position

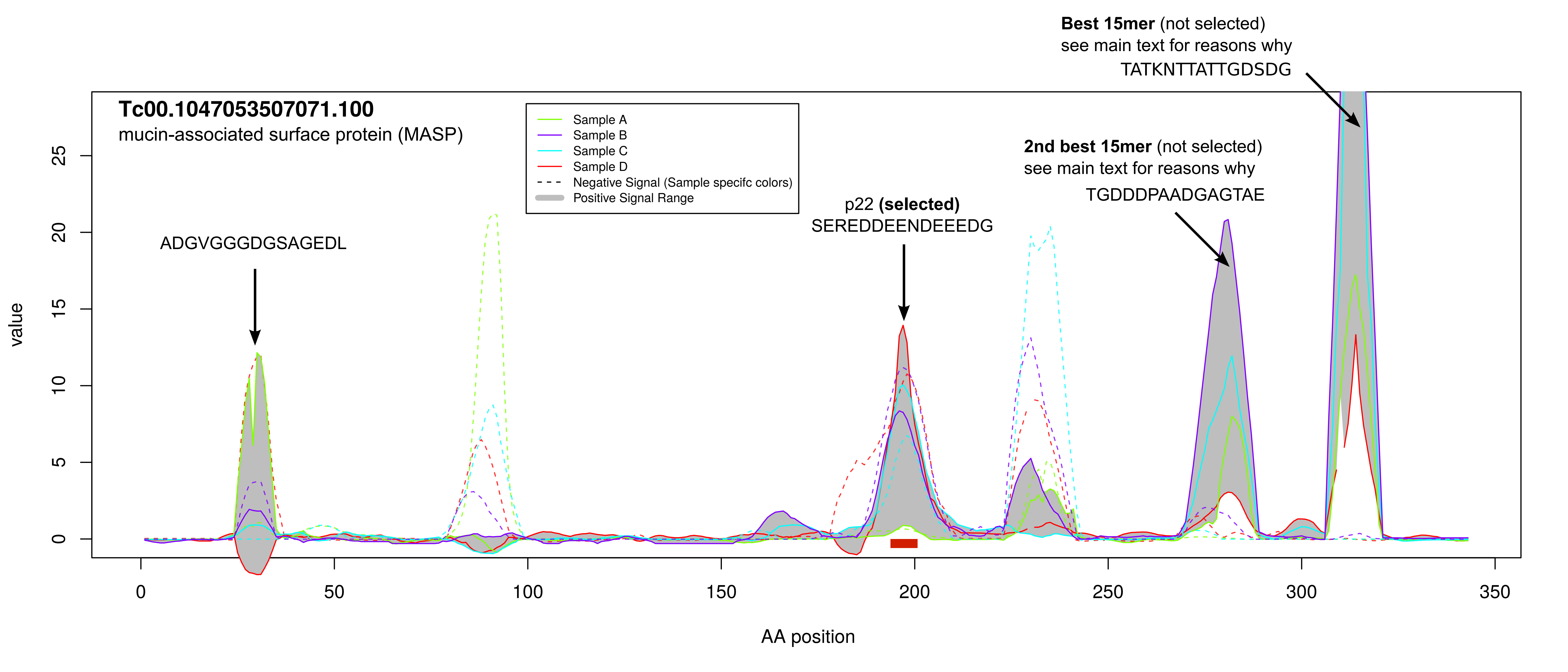
Best 15mer (not selected)
see main text for reasons why
TATKNTTATTGDSDG
Tc00.1047053507071.100
mucin-associated surface protein (MASP)
Sample A
Sample B
Sample C
Sample D
Negative Signal (Sample specifc colors)
Positive Signal Range
2nd best 15mer (not selected)
see main text for reasons why
TGDDDPAADGAGTAE
p22 (selected)
SEREDDEENDEEEDG
ADGVGGGDGSAGEDL
value
AA position
0
5
10
15
20
25
0
50
100
150
200
250
300
350

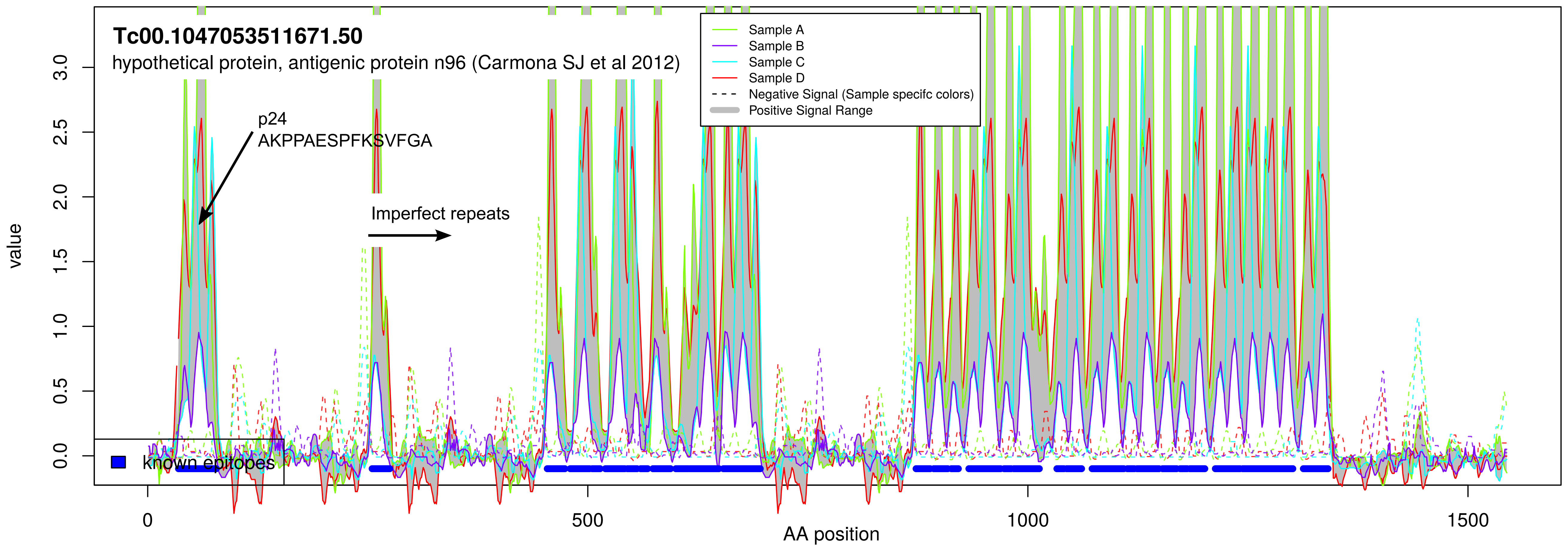
Tc00.1047053511671.50
hypothetical protein, antigenic protein n96 (Carmona SJ et al 2012)
Sample A
Sample B
Sample C
Sample D
Negative Signal (Sample specifc colors)
Positive Signal Range
p24
AKPPAESPFKSVFGA
Imperfect repeats
known epitopes
value
AA position
0
500
1000
1500
0.0
0.5
1.0
1.5
2.0
2.5
3.0

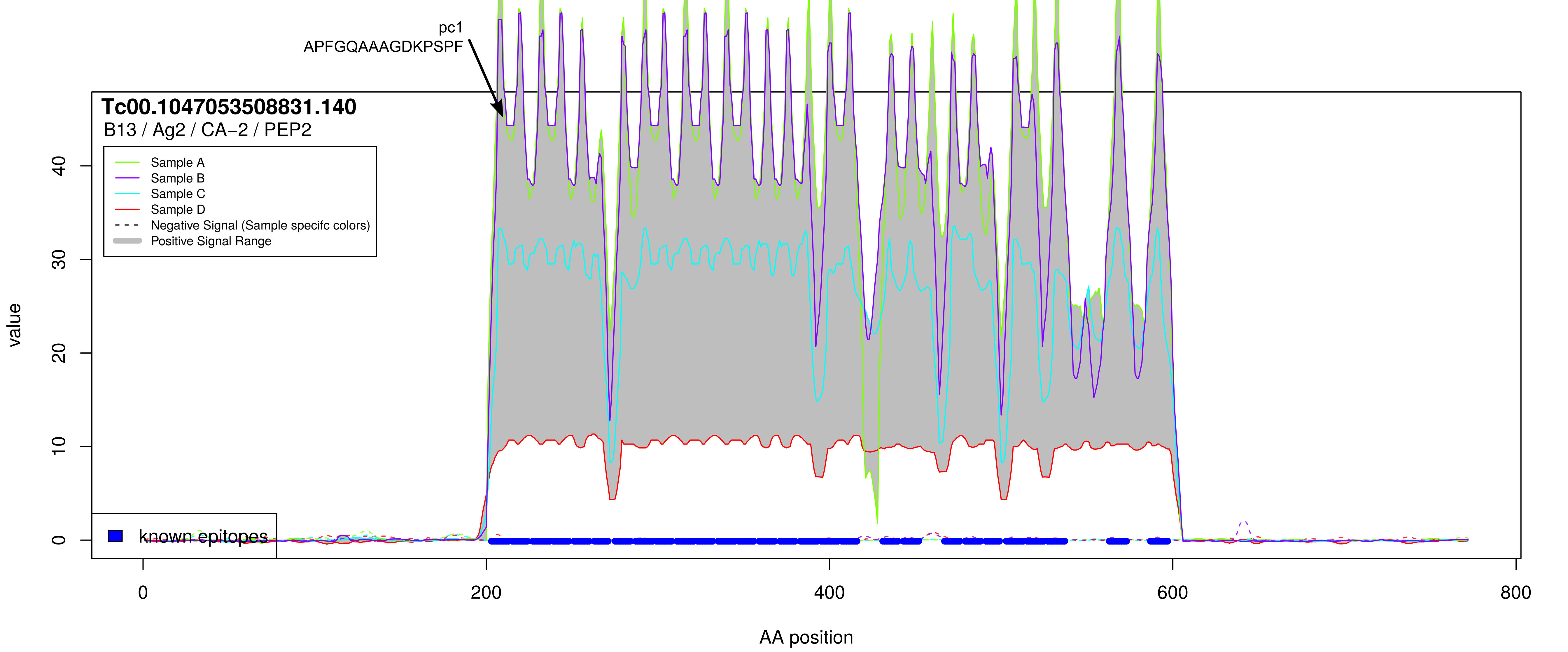

pc1
APFGQAAAGDKPSPF
Tc00.1047053508831.140
B13 / Ag2 / CA−2 / PEP2
Sample A
Sample B
Sample C
Sample D
Negative Signal (Sample specifc colors)
Positive Signal Range
known epitopes
value
AA position
0
10
20
30
40
0
200
400
600
800

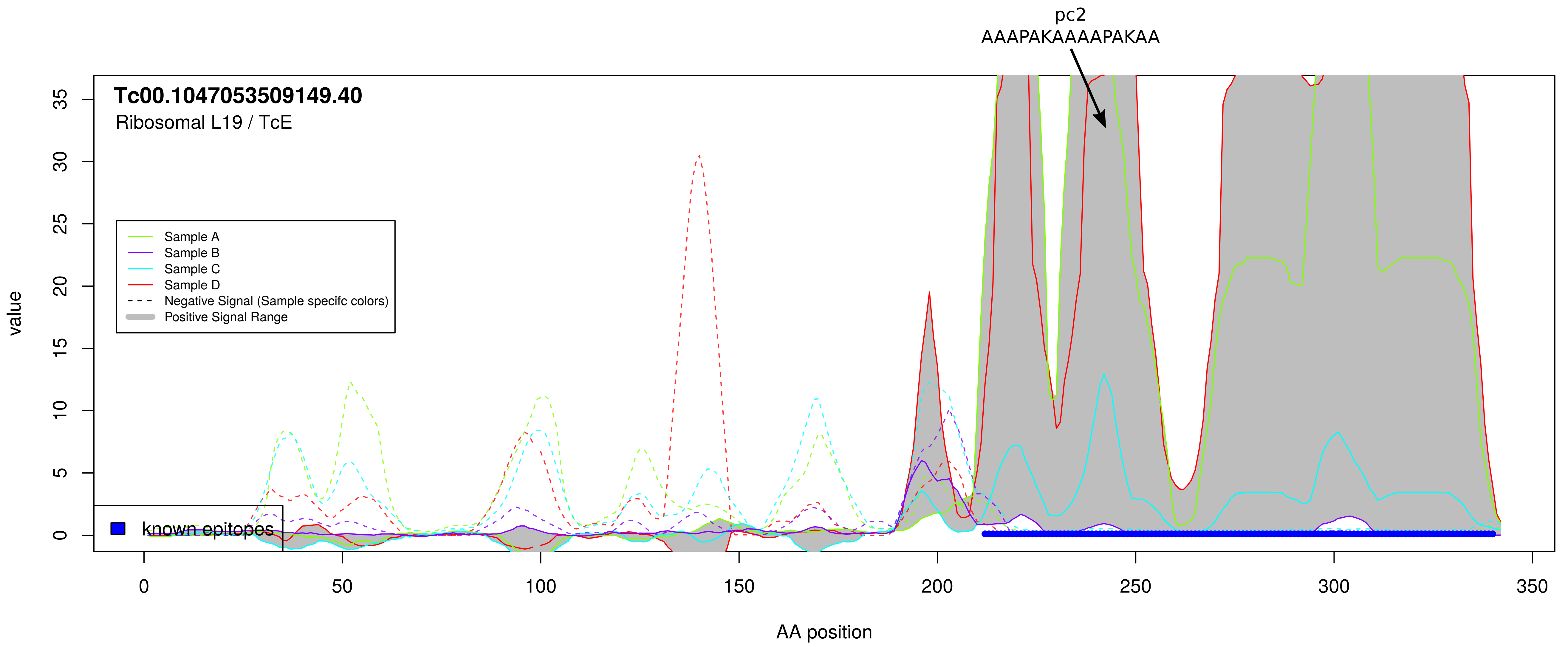

Tc00.1047053509149.40
Ribosomal L19 / TcE
pc2
AAAPAKAAAAPAKAA
Sample A
Sample B
Sample C
Sample D
Negative Signal (Sample specifc colors)
Positive Signal Range
known epitopes
value
AA position

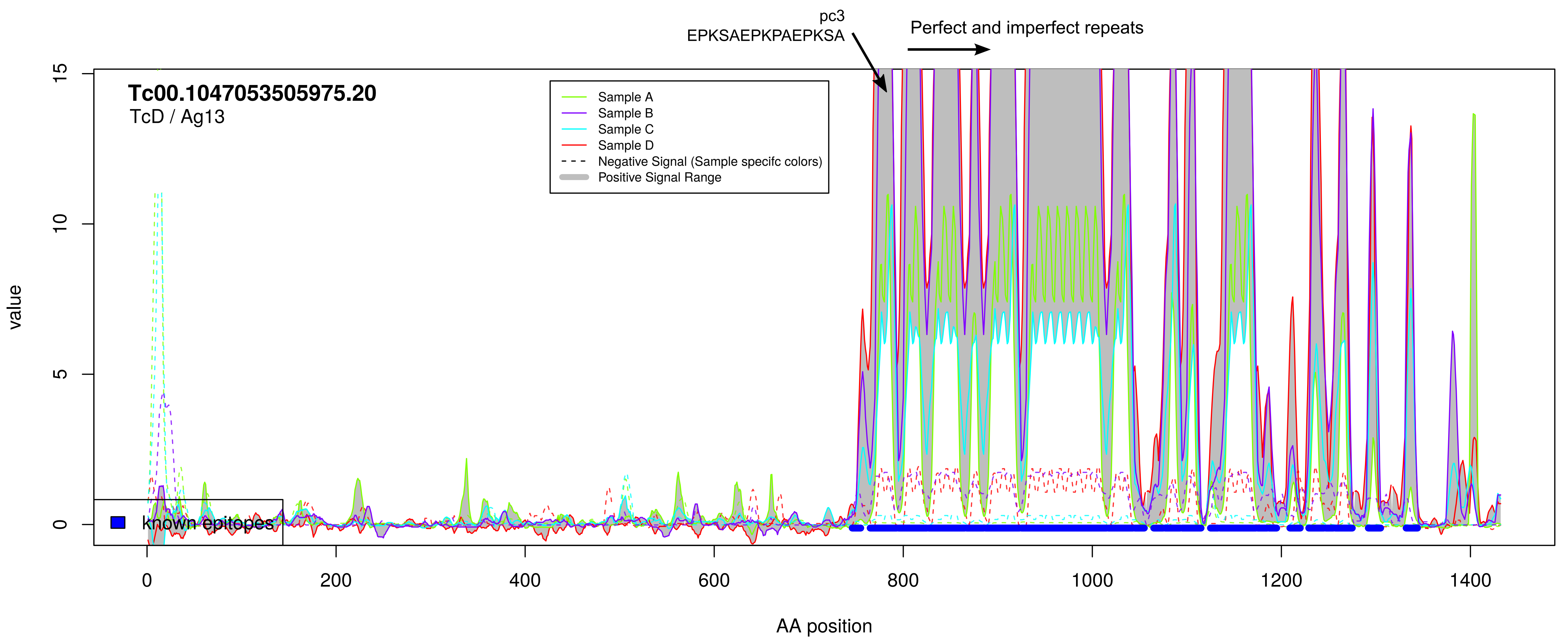
pc3
EPKSAEPKPAEPKSA
Perfect and imperfect repeats
Tc00.1047053505975.20
TcD / Ag13
Sample A
Sample B
Sample C
Sample D
Negative Signal (Sample specifc colors)
Positive Signal Range
known epitopes
value
AA position
0
5
10
15
0
200
400
600
800
1000
1200
1400
