# Supplementary figures and images for: Next-generation ELISA diagnostic assay for Chagas Disease based on the combination of short peptidic epitopes
Source: PLoS Negl Trop Dis. 2017 Oct 9;11(10):e0005972. doi: 10.1371/journal.pntd.0005972 (PMC5648266; doi:10.1371/journal.pntd.0005972)

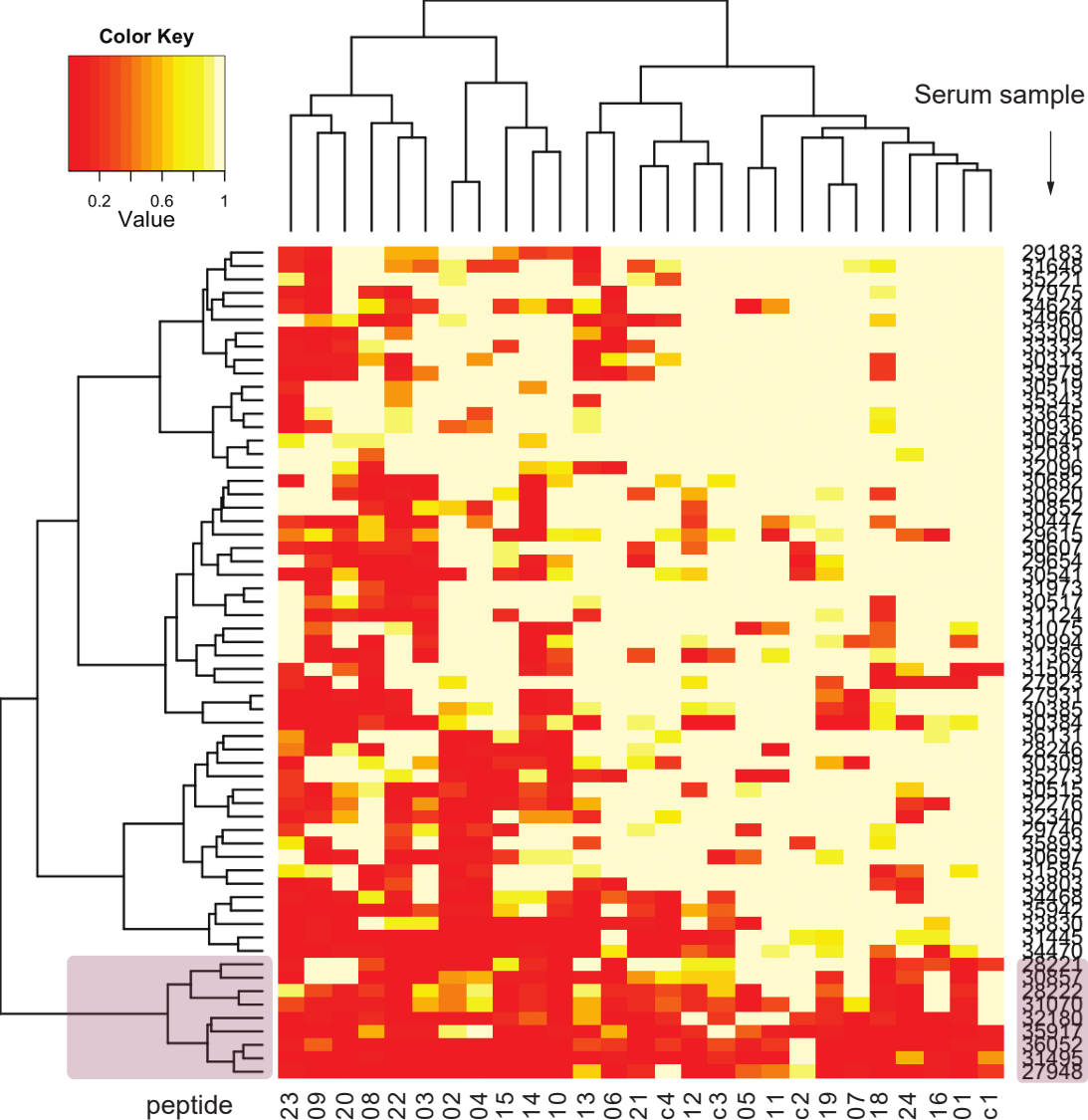

Supplement: S2 Fig — Heatmap display of ELISA reactivity of each of the 27 peptides tested against a panel of 62 positive sera samples. For the heatmap display the reactivity values (in the form of z-scores above background) were transformed for clarity using a sigmoid function centered around 3. Peptides and subjects were clustered using a hierarchical clustering algorithm (R, hclust). A group of subjects showing moderately low ELISA reactivity across peptides has been highlighted (see main text). File: S2 Fig. (PDF) [file pntd.0005972.s002.pdf]

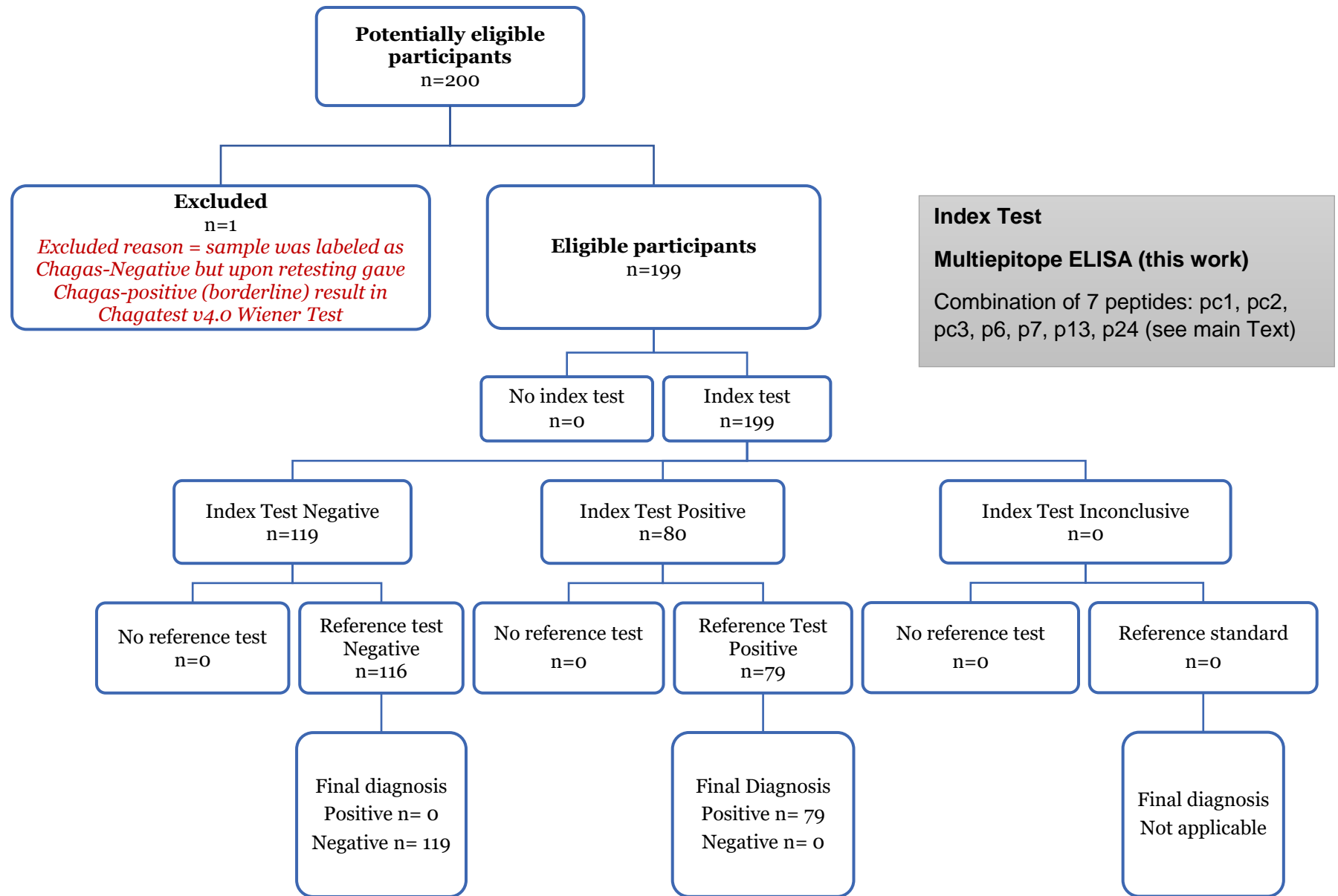

Supplement: S3 Fig — (PDF) [file pntd.0005972.s003.pdf]
