# Supplementary material for: Next-generation ELISA diagnostic assay for Chagas Disease based on the combination of short peptidic epitopes
Source: PLoS Negl Trop Dis. 2017 Oct 9;11(10):e0005972. doi: 10.1371/journal.pntd.0005972 (PMC5648266; doi:10.1371/journal.pntd.0005972)
Supplement: S2 Table — (PDF) [file pntd.0005972.s005.pdf]

S2 Table – STARD Checklist for reporting studies of diagnostic accuracy

Section & Topic	No	Item	Reported on page #
**TITLE OR ABSTRACT**			
	**1**	Identification as a study of diagnostic accuracy using at least one measure of accuracy (such as sensitivity, specificity, predictive values, or AUC)	2, Abstract
**ABSTRACT**			
	**2**	Structured summary of study design, methods, results, and conclusions (for specific guidance, see STARD for Abstracts)	2, Abstract
**INTRODUCTION**			
	**3**	Scientific and clinical background, including the intended use and clinical role of the index test	4, Introduction
	**4**	Study objectives and hypotheses	4, Introduction
**METHODS**			
Study design	**5**	Whether data collection was planned before the index test and reference standard were performed (prospective study) or after (retrospective study)	7,8<br>Retrospective Study
Participants	**6**	Eligibility criteria	7,8
	**7**	On what basis potentially eligible participants were identified (such as symptoms, results from previous tests, inclusion in registry)	7,8
	**8**	Where and when potentially eligible participants were identified (setting, location and dates)	7,8
	**9**	Whether participants formed a consecutive, random or convenience series	7,8<br>Convenience series
Test methods	**10a**	Index test, in sufficient detail to allow replication	6 (Table 1), 13 Results (final peptide combination)
	**10b**	Reference standard, in sufficient detail to allow replication	7,8 (Chagatest v4.0 Wiener Labs)
	**11**	Rationale for choosing the reference standard (if alternatives exist)	16, Discussion Availability in Argentina, widespread use elsewhere
	**12a**	Definition of and rationale for test positivity cut-offs or result categories of the index test, distinguishing pre-specified from exploratory	9, Methods, Data Analysis
	**12b**	Definition of and rationale for test positivity cut-offs or result categories of the reference standard, distinguishing pre-specified from exploratory	Brochure of the manufacturer
	**13a**	Whether clinical information and reference standard results were available to the performers/readers of the index test	7,8 (Methods)<br>YES
	**13b**	Whether clinical information and index test results were available to the assessors of the reference standard	NO
Analysis	**14**	Methods for estimating or comparing measures of diagnostic accuracy	9 (Methods, Data analysis)
	**15**	How indeterminate index test or reference standard results were handled	9 (Methods, Data analysis)<br>No indeterminates
	**16**	How missing data on the index test and reference standard were handled	Not applicable
	**17**	Any analyses of variability in diagnostic accuracy, distinguishing pre-specified from exploratory	
	**18**	Intended sample size and how it was determined	8 (Methods)<br>>73 Chagas positive samples<br>>73 Chagas-negative samples
**RESULTS**			
Participants	**19**	Flow of participants, using a diagram	S3 Figure
	**20**	Baseline demographic and clinical characteristics of participants	
	**21a**	Distribution of severity of disease in those with the target condition	7,8 (Methods)

CC BY SA

S2 Table – STARD Checklist for reporting studies of diagnostic accuracy

			Chagas (n=80) all asymptomatic without cardiac or gastrointestinal compromise.
	**21b**	Distribution of alternative diagnoses in those without the target condition	Not applicable
	**22**	Time interval and any clinical interventions between index test and reference standard	Not applicable. Samples from sera bank collection
Test results	**23**	Cross tabulation of the index test results (or their distribution) by the results of the reference standard	S1 Table
	**24**	Estimates of diagnostic accuracy and their precision (such as 95% confidence intervals)	13, 14; Table 3
	**25**	Any adverse events from performing the index test or the reference standard	None
**DISCUSSION**			
	**26**	Study limitations, including sources of potential bias, statistical uncertainty, and generalisability	17
	**27**	Implications for practice, including the intended use and clinical role of the index test	Proof of concept, Feasibility study
**OTHER INFORMATION**			
	**28**	Registration number and name of registry	Not applicable
	**29**	Where the full study protocol can be accessed	7
	**30**	Sources of funding and other support; role of funders	In submission form

CC BY SA
